# Deciphering the Adaptation of *Corynebacterium glutamicum* in Transition from Aerobiosis via Microaerobiosis to Anaerobiosis

**DOI:** 10.3390/genes9060297

**Published:** 2018-06-13

**Authors:** Julian Lange, Eugenia Münch, Jan Müller, Tobias Busche, Jörn Kalinowski, Ralf Takors, Bastian Blombach

**Affiliations:** 1 Institute of Biochemical Engineering, University of Stuttgart, Allmandring 31, 70569 Stuttgart, Germany; julian.lange@web.de (J.L.); eugenia.muench@ibvt.uni-stuttgart.de (E.M.); Jan-Mueller1992@web.de (J.M.); takors@ibvt.uni-stuttgart.de (R.T.); 2 Center for Biotechnology, Bielefeld University, Universitätsstraße 27, 33615 Bielefeld, Germany; tbusche@cebitec.uni-bielefeld.de (T.B.); joern@cebitec.uni-bielefeld.de (J.K.); 3 Institute for Biology-Microbiology, Freie Universität Berlin, Königin-Luise-Str. 12-16, 14195 Berlin, Germany

**Keywords:** *Corynebacterium glutamicum*, transcriptional response, aerobiosis, microaerobiosis, anaerobiosis, triple-phase process

## Abstract

Zero-growth processes are a promising strategy for the production of reduced molecules and depict a steady transition from aerobic to anaerobic conditions. To investigate the adaptation of *Corynebacterium glutamicum* to altering oxygen availabilities, we conceived a triple-phase fermentation process that describes a gradual reduction of dissolved oxygen with a shift from aerobiosis via microaerobiosis to anaerobiosis. The distinct process phases were clearly bordered by the bacteria’s physiologic response such as reduced growth rate, biomass substrate yield and altered yield of fermentation products. During the process, sequential samples were drawn at six points and analyzed via RNA-sequencing, for metabolite concentrations and for enzyme activities. We found transcriptional alterations of almost 50% (1421 genes) of the entire protein coding genes and observed an upregulation of fermentative pathways, a rearrangement of respiration, and mitigation of the basic cellular mechanisms such as transcription, translation and replication as a transient response related to the installed oxygen dependent process phases. To investigate the regulatory regime, 18 transcriptionally altered (putative) transcriptional regulators were deleted, but none of the deletion strains showed noticeable growth kinetics under an oxygen restricted environment. However, the described transcriptional adaptation of *C. glutamicum* resolved to varying oxygen availabilities provides a useful basis for future process and strain engineering.

## 1. Introduction

*Corynebacterium glutamicum* is an established workhorse in industrial biotechnology and is used for the production of amino acids such as monosodium glutamate (MSG) and l-lysine with a market size of 3.1 and 2.2 million tons per year in 2015 [1]. Furthermore it has also been exploited for the synthesis of a variety of other fuels and chemicals [2,3]. Especially, the facultatively anaerobic lifestyle of this Gram-positive bacterium [4], formed the basis to engineer *C. glutamicum* for the production of reduced molecules such as organic acids (e.g., lactate, succinate) and alcohols (e.g., ethanol, isobutanol) under zero-growth anaerobic conditions [5,6,7,8,9].

Zero-growth production processes often start with an aerobic phase for biomass formation, which is accompanied by an anaerobic production phase with resting cells either in separated vessels (two-stage process) or in one reactor as a dual-phase process [10]. However, a common challenge in the development of zero-growth production processes is that fast transitions from aerobiosis to anaerobiosis, as prevalent in dual-phase approaches, might lead to deficiencies in cell viability, the product yield and production rates [10]. Interestingly, applying triple-phase processes, which additionally provide an oxygen-limited interface, led to a (partial) restoration of the performance in the successive anaerobic production phase [11,12,13,14]. For example, it was shown with a lactate dehydrogenase-deficient strain of *C. glutamicum* that a progressive deoxygenation enhanced succinate and acetate titers by up to 640% [15]. The beneficial effect was attributed to the low aerated intermediate state, often referred to as microaerobiosis. Obviously, this phase plays an essential role in the physiological adaptation and preparation of the enzymatic machinery to complete anaerobic conditions [7,8,13]. However, microaerobiosis is also discussed to negatively impact cell viability in large-scale bioreactors, where bacteria face changing oxygen availabilities due to insufficient power input and mixing [16,17,18]. Such fluctuations might go hand in hand with reduced productivities and product yields [18,19,20,21].

With respect to current knowledge, microaerobiosis has been insufficiently defined and is difficult to distinguish from the aerobic and anaerobic phase. Currently, microaerobiosis is mostly referred to as low dissolved oxygen concentrations (DO) conditions between 0–5% [5,11,13,22,23,24,25,26]. More explicitly, Kaboré et al. [15] defined microaerobic conditions by constantly limiting oxygen transfer rates and used this definition as a process control for enhanced succinate and acetate production in *C. glutamicum*. Microaerobiosis was also characterized in situ by the determination of metabolic states via online fluorescence of NAD(P)H of denitrifying *Pseudomonas aeruginosa* [27]. Indirect process control by redox probes to analyze the oxygen-reduction potential (ORP) is an established method in the wastewater treatment processes [28,29]. Such redox probes were also applied to monitor two-stage [30] and dual-phase [31] processes. Alternatively, oxygen limitation can also be described using Michaelis–Menten constants (K*_S_*-values), which directly link oxygen availability to the specific growth rate [32]. Microaerobiosis can thus be defined under submaximal growth rates with oxygen being the sole limiting substrate.

Although metabolic engineering tools and omics technologies for systems level analysis of *C. glutamicum* are available and significantly contributed to the current knowledge of the regulatory repertoire [33,34,35,36,37,38,39], the understanding of the oxygen-related adaptation and its regulation is still limited. In *Escherichia coli* known key players of oxygen-dependent regulation were identified and harness a directly oxygen sensing iron-sulfur cluster protein FNR [40,41], the two-component systems ArcBA [42] and DipB/DipA [43] and the chemotaxis system Aer [44]. As dual-regulator, FNR directly senses molecular oxygen, activates genes of the anaerobic metabolism and inhibits functions involved in aerobic respiration [45]. ArcB and ArcA form a two-component system, where ArcB senses the redox state of the quinone pool in the membrane and phosphorylates the cognate response regulator ArcA in the absence of oxygen [46]. The interplay between FNR and ArcBA allows an oxygen-dependent fine tuning of the cellular metabolism [47,48]. Furthermore, the metabolic flux distributions are influenced by intracellular metabolite concentrations and cofactor availability such as NADH or NAD^+^ [49]. For *E. coli* mechanistic models at systems-level for the FNR cycle at transitions from aerobiosis to anaerobiosis and the general response towards oxygen are available in literature [50,51]. Such a comprehensive picture about the oxygen-related regulatory and metabolic network is, so far, not available for *C. glutamicum*. Previous works describe the physiological adaptation with transcriptional profiling to a shift from aerobiosis to complete anaerobiosis [30,52] also with respect to a genome wide metabolic model verification [53]. More recent studies addressed the impact of large scale inhomogeneities with regard to altering oxygen availabilities on the metabolism of *C. glutamicum* under scale-down conditions [19,54,55,56] and aimed to resolve the cellular adaptation events in the interval of the mixing time (~3 min) of a production bioreactor [57,58,59].

In contrast to the described approaches, we established a triple-phase process that mirrors a typical zero-growth approach [10] and therefore depicts a gradual shift from aerobiosis to anaerobiosis and provides a defined microaerobic interface. The observed distinct physiological characteristics served as useful criterium to delineate each process phase. The analysis of the transient transcriptional adaptation to the applied increasing oxygen-limitation disclosed an early response with the onset of microaerobiosis to coordinate aerobic respiration and fermentation during growth in parallel and a late response upon strict anaerobic conditions to prime the metabolism for non-growing conditions.

## 2. Materials and Methods

### 2.1. Bacterial Strain and Media

*C. glutamicum* ATCC 13032 was purchased from the American Type Culture Collection and was cultivated in 2× yeast extract tryptone (YT) complex medium [60] and modified CGXII minimal medium based on literature [61,62]. The medium contained per liter 5 g (NH_4_)_2_SO_4_, 5 g urea, 21 g 3-(N-morpholino) propane sulphonic acid (MOPS), 1 g KH_2_PO_4_, 1 g K_2_HPO_4_, 0.25 g MgSO_4_·7 H_2_O, 10 mg CaCl_2_, 10 mg MnSO_4_·H_2_O, 16.4 mg FeSO_4_·7 H_2_O, 1 mg ZnSO_4_·7 H_2_O, 0.2 mg CuSO_4_·5 H_2_O, 0.02 mg NiCl_2_·6 H_2_O, 0.2 mg biotin, and 30 mg protocatechuate (PCA). For cultivation, a pH of 7.4 was used based on the analyzed intracellular value [63]. For bioreactor cultivations, the medium lacked urea and MOPS and is referred to as CGXII*. As carbon source, d-glucose was added from a 500 g L^−1^ aqueous stock solution as indicated. Long time storage of bacterial strains was achieved at −70 °C in 30% (*v*/*v*) glycerol.

### 2.2. Cultivation Conditions

*General*. *C. glutamicum* was cultivated at 30 °C in a bioreactor or in shaking flasks on a rotary shaker at 120 rpm agitation.

*Aerobic/microaerobic shaking flasks*. Bacterial suspensions were cultivated in 500 mL flasks with four baffles. The subsequent seed train was followed (cultivation condition/inoculum/incubation time): 2× YT agar plates/streaked from glycerol stock/2–3 days; overday (o/d) culture in 5 mL 2× YT/single colony/6–8 h; overnight (o/n) culture in 50 mL 2× YT/complete o/d culture/12–16 h; main culture in 50 mL CGXII + 60 g glucose L^−1^/to desired biomass concentration from o/n culture. For inoculation of the main culture to an appropriate amount of cells from the o/n cultivation was harvested by centrifugation (4500 rcf for 5–10 min, centrifuge 5804 R, rotor: A-4-44, Eppendorf AG, Hamburg, Germany), resuspended in 0.9% (*w*/*v*) NaCl solution and added aseptically.

*Anaerobic shaking flasks*. The cultivation was performed in sealed and unbaffled 100 mL flasks including a silicon septum in the lid that prevents gas exchange and enables aseptic inoculation and sampling through syringes. The following seed train was used (cultivation condition/inoculum/incubation time): 2× YT agar plates/streaked from glycerol stock/2–3 days; o/n_1_ in 5 mL 2× TY/single colony/15–16 h; o/d in 50 mL 2× YT/complete o/n_1_/7–8 h; o/n_2_ in 50 mL CGXII + 40 g glucose L^−1^/o/d (starting biomass ~0.2 g CDW L^−1^)/13–14 h; anaerobic culture in an anaerobic flask with 50 mL CGXII + 20 g glucose L^−1^/o/n_2_ (starting biomass ~3.7 g CDW L^−1^). Anaerobic flasks were flushed for 10 min with N_2_ prior to inoculation through a syringe. After 4 h of cultivation, cells were harvested and protein and enzymatic analysis was conducted as described below.

*Triple-phase bioprocess*. To investigate the adaptation of *C. glutamicum* to a transition from aerobiosis via microaerobiosis to anaerobiosis a bioprocess in a 30 L stainless steel bioreactor (Bioengineering AG, Wald, Switzerland) was conceptualized. The seed train was established as follows (cultivation condition/inoculum/incubation time): 2× TY agar plate/streaked from glycerol stock/2–3 days; o/n_1_ in 5 mL 2× TY/single colony/15–16 h; o/d in 50 mL 2× YT/complete o/n_1_/7–8 h; o/n_2_ in 200 mL CGXII + 40 g glucose L^−1^ in 2 L flasks with baffles/o/d (starting biomass ~0.2 g CDW L^−1^)/11–12 h; main culture in 10 L CGXII* + 60 g glucose L^−1^ in the 30 L bioreactor/o/n_2_ (starting biomass ~0.4 g CDW L^−1^). The online pH and dissolved oxygen (DO) were measured using standard probes (Mettler-Toledo GmbH, Gießen, Germany). Calibration was achieved ex situ at 30 °C for the DO electrode at 0% (dH_2_O + sodium sulfite) and 100% (dH_2_O + air gassing) and pH electrodes in buffer solutions at pH 4.0 and pH 7.0. To maintain a pH of 7.4 throughout the cultivation, 25% ammonia solution was added automatically. Excessive foaming was avoided by adding anti-foam solution manually on demand (Struktol™ J 647, Schill + Seilacher GmbH, Hamburg, Germany). Over the entire cultivation period the agitation was set to 445 rpm using a single six blade Rushton turbine and four baffles at the reactor wall. Low aeration rates of 0.1 vvm (1 L min^−1^; mass flow controller 0–2 L min^−1^, Analyt-MTC GmbH, Müllheim, Germany) were realized by gassing through a needle inside the cultivation broth. The exhaust gas was analyzed for O_2_ and CO_2_ content (BlueSens gas sensor GmbH, Herten, Germany) and the reactor overpressure kept at 0.5 bar. After 11 h of cultivation anaerobic conditions were initiated by a stop of aeration and flushing the headspace with 10 L nitrogen gas min^−1^ for 15 min. Under anaerobic conditions no exhaust gas analysis was conducted.

### 2.3. Analytical Methods

#### 2.3.1. Optical Density

Bacterial growth was monitored by photometric inspection of turbidity (optical density, OD). A biosuspension sample was for this purpose diluted in 0.9% (*w*/*v*) NaCl and analyzed at 600 nm wavelength (OD_600_) via the Ultrospec 10 Cell Density Meter (GE Healthcare Europe GmbH, Freiburg, Germany) or DR 2800 Portable spectrophotometer (Hach Lange GmbH, Düsseldorf, Germany) in the range of 0.1–0.3.

#### 2.3.2. Cell Dry Weight

Analysis of the cell dry weight (CDW) was achieved with the following procedure. Test tubes were dried at 105 °C for >1 day, cooled to room temperature (RT) in a desiccator and weighed (centrifuge tubes round bottom DURAN^®^, 12 mL, Carl-Roth GmbH, Karlsruhe, Germany). From a cultivation experiment, 5 mL biosuspension were taken and harvested in the test tubes (4000 rcf, 10 min, 4 °C; centrifuge 5427 R, rotor: F-35-6-30, Eppendorf AG). After discarding the supernatant, the pellet was washed twice with 5 mL dH_2_O and dried for >2 days at 105 °C. Finally, the CDW was analyzed differentially. The OD_600_ was correlated to the CDW concentrations by the coefficients α (8-level CDW/OD_600_ correlation curves). These were representatively determined with *C. glutamicum* in CGXII* medium and 60 g glucose L^−1^ as sole substrate over a broad range of growth rates (0.02–0.47 h^−1^). An α of 0.22 g L^−1^ and 0.30 g L^−1^ per OD was calculated for the respective photometers (Ultrospec 10 Cell Density Meter, GE Healthcare and DR 2800 Spectrophotometer, Hach Lange GmbH).

#### 2.3.3. Enzyme Assays

*Lysates*. Bacterial lysates for enzyme assays with *C. glutamicum* were produced from aerobic and anaerobic shaking flask cultivations. For this purpose, the cells were harvested by centrifugation (4500 rcf, 10 min; centrifuge 5804 R, rotor: A-4-44, Eppendorf AG). A pellet of approximately 50 mg CDW was subsequently washed once in 0.2 M Tris-HCl buffer (pH 7.4), resuspended in 400 µL lysis buffer (0.1 M Tris-HCl (pH 7.4), 10% (*v*/*v*) glycerol) and added to ~300 µL glass beads in cryo reaction tubes. Cell disruption occurred mechanically in a Precellys^®^24 apparatus (Bertin Instruments, Montigny-le-Bretonneux, France) at 3× 20 s (6500 rpm) cycles. The soluble extract was purified from cell debris by centrifugation (20,000 rcf, 1 min, 4 °C; centrifuge 5804 R, rotor: FA 45-30-11, Eppendorf AG). Lysate supernatants were stored on ice until further analysis.

*Enzyme activities*. Activities of glucose-6-phosphate dehydrogenase (G6P-DH) and 6-phosphogluconate dehydrogenase (6PG-DH) were determined in clarified bacterial lysates following a modified protocol from Moritz et al. [64]. For this purpose, photometric analysis at 340 nm wavelength (ε = 6.3 (mM cm)^−1^) was conducted in acrylic semi-micro cuvettes (diameter 1 cm, Sarstedt AG & Co, Nümbrecht, Germany) using an Ultrospec™ 2100 pro UV/Visible spectrophotometer (GE Healthcare) at 30 °C. The reaction batch constituted: 500 µL analysis buffer (50 mM Tris-HCl (pH 7.5), 10 mM MgCl_2_ and 1 mM NADP^+^), 50–100 µL 1:10 diluted cell extract, and dH_2_O added to 0.95 mL assay volume. The extinction was followed for 1 min prior to the start of reaction by addition of 50 µL 4 mM glucose-6-phosphate or 1 mM 6-phospho-gluconate for the analysis of G6P-DH or 6PG-DH activity, respectively. The increase of extinction was then followed for 5 min. The slopes in both phases were determined by linear regression and the rectified volumetric enzyme activity (U mL^−1^) was calculated after Beer–Lambert’s law. The specific activity (U mg_protein_^−1^) was eventually derived by division of the volumetric activity by the soluble protein concentration of the bacterial lysate.

#### 2.3.4. Protein Quantification

Bacterial lysates of *C. glutamicum* were analyzed following the manufacturer’s instructions (*BCA Protein Assay Kit*, Thermo Fisher Scientific Inc., Waltham, MA, USA). Soluble protein quantification was achieved by analyzing a 9-level standard calibration curve of diluted bovine serum albumin (BSA) at 560 nm using a Synergy 2 microplate reader (BioTek Instruments GmbH, Bad Friedrichshall, Germany) and 96-well plates (Greiner Bio-One GmbH, Frickenhausen, Germany).

#### 2.3.5. High-Performance Liquid Chromatography

*d-Glucose and organic acids*. Bacterial suspension samples were treated by centrifugation (≥11,000 rcf, ≥1 min; Centrifuge MiniSpin^®^, rotor F-45-12-11, Eppendorf AG) to yield clarified culture supernatants. In these samples, concentrations of metabolites (glucose, acetate, lactate, succinate) were quantified according to literature [65] using an Agilent 1200 series apparatus (Agilent Technologies, Santa Clara, CA, USA) equipped with a Rezex^TM^ ROA-Organic Acid H^+^ (8%) LC column (300 × 7.8 mm, 8 µm) protected by a Carbo-H^+^ SecurityGuard™ (4 × 3 mm) column (Phenomenex Inc., Aschaffenburg, Germany). Supernatant pretreatment was conducted according to Buchholz et al. [65] to facilitate a precipitation of phosphate. 45 µL 4 M NH_3_ and 100 µL 1.2 M MgSO_4_ were added to 1 mL supernatant. After 5 min of incubation, the sample was centrifuged (5 min, 18,000 rcf, RT; centrifuge 5417 R, rotor: FA45-30-11, Eppendorf AG). 500 µL the supernatant were subsequently transferred to 500 µL 0.1 M H_2_SO_4_, mixed and incubated (15 min, RT). Finally, a clarification was performed by centrifugation (18,000 rcf, 15 min, RT). Isocratic chromatography was realized with 5 mM H_2_SO_4_ as mobile phase and 0.4 mL min^−1^ flow rate for 45 min at 50 °C column temperature. 10 µL of the pretreated supernatant were injected to the sample loop and detection achieved with an Agilent 1200 series refractive index detector at 32 °C. Peaks were quantified using 6-level standard calibrations for each analyte as external reference. To account for pretreatment variabilities, l-rhamnose was spiked as internal standard prior to phosphate precipitation.

*l-Glutamate*. Intracellular l-glutamate concentrations were analyzed in chemical lysates of *C. glutamicum* within the triple-phase process from samples ①–⑥. Lysis was achieved with perchloric acid using a modified procedure from literature [66,67]. 1 mL of bacterial suspension (2–5 g CDW L^−1^) was pipetted to 0.25 mL lysis buffer (−20 °C, 35% (*v*/*v*) perchloric acid, 80 µM ethylenediaminetetraacetic acid (EDTA) and incubated for equilibration (rolling, 15 min, 4 °C). Then, 0.25 mL 1 M K_2_HPO_4_ was added and stepwise neutralization to pH 7.0 was achieved with 5 M KOH. After centrifugation (20,000 rcf, 5 min, 4 °C; centrifuge 5427 R, rotor: FA 45-30-11, Eppendorf AG), the pH was verified and the sample stored at −70 °C until measurement. High-performance liquid chromatography (HPLC) analysis of intracellular l-glutamate concentrations within the lysates was achieved in an Agilent 1200 series apparatus equipped with an Agilent Zorbax Eclipse Plus C18 (250 × 4.6 mm, 5 µm) column protected by an Agilent Zorbax Eclipse Plus C18 (12.5 × 4.6 mm, 5 µm) guard column (Agilent Technologies) as described previously [65]. A pre-column derivatization with *o*-phthaldialdehyde (OPA) and fluorometric detection, with excitation at 230 nm and emission at 450 nm was realized. For elution, the buffer consisted of a polar phase (10 mM Na_2_HPO_4_, 10 mM Na_2_B_4_O_7_, 0.5 mM NaN_3_, pH 8.2) and a nonpolar phase (45% (*v*/*v*) methanol, 45% (*v*/*v*) acetonitrile). The injection volume was 36.4 µL (2.5 µL 10 mM Na_2_B_4_O_7_, 1 µL sample, 0.5 µL OPA reagent, 0.4 µL Fmoc chloride and 32 µL injection dilution solution). Quantification was accomplished analyzing a 7-point l-glutamate calibration curve as external standard. Variabilities in the procedure were corrected by implementation of a 200 mM l-ornithine internal standard. To recalculate the analyses to intracellular concentrations, the following considerations were followed: First, the analyzed concentration was recalculated to the utilized biomass within the harvested sample and taking lysis dilution effects into account. Second, the published cell volume to biomass ratio of 1.95 μL per mg CDW by Krömer et al. was harnessed to determine the intracellular titer [68].

### 2.4. RNA-Sequencing

#### 2.4.1. Sample Harvest and RNA Isolation

Throughout the triple-phase process, six sequential biomass samples were harvested in a fast sampling procedure at various growth rates and oxygen availabilities. For this purpose, approximately 2 × 10^9^ cells of cell suspension were filled into precooled (−21 °C) 2 mL reaction tubes. Pellets were then harvested in an immediate centrifugation (≥13,000 rcf, 30 s; centrifuge MiniSpin^®^, rotor F-45-12-11 (pre-cooled to −21 °C), Eppendorf AG). Then, the supernatant was discarded, the cell pellets frozen in liquid N_2_ and stored at −70 °C. RNA isolation was performed using the RNeasy Mini Kit (Qiagen, Hilden, Germany) including a double DNA digestion step. RNA quality and quantity was checked by using NanoDrop (Peqlab, VWR, Radnor, PA, USA) and by Agilent RNA Nano 6000 kit on Agilent 2100 Bioanalyzer (Agilent Technologies). PCR was performed to assure that no DNA remained in the samples.

#### 2.4.2. Complementary DNA Library Preparation and Sequencing

Four micrograms of total RNA with a RNA integrity number (RIN) >8.0 was used for complementary DNA (cDNA) library preparation. Stable ribosomal RNAs (rRNAs) were depleted with the Ribo-Zero rRNA Removal Kit (Bacteria) according to the manufacturer’s instructions (Epicentre, Madison, WI, USA). Afterwards, the remaining messenger RNA (mRNA) was purified using RNA MinElute columns (Qiagen) and checked for quality and successful rRNA depletion with the Agilent RNA Pico 6000 kit on the Agilent 2100 Bioanalyzer (Agilent Technologies). The TruSeq Stranded mRNA Library Prep Kit from Illumina was applied to prepare the cDNA libraries. The cDNAs were sequenced paired end on an Illumina MiSeq system using 75 nt read length. The transcriptome sequencing raw data files are available in the ArrayExpress database (www.ebi.ac.uk/arrayexpress) under accession number: E-MTAB-6602.

#### 2.4.3. Data Analysis, Read Mapping, Data Visualization and Analysis of Differential Gene Expression

The sequenced cDNA reads were trimmed for low quality bases from both ends and a sliding window trimming (removing bases when the average quality per base in a window of 4 nt decreases below 15) using trimmomatic v0.36 [69]. Reads with a minimal length of 36 nt were mapped with bowtie2 v2.2.7 [70] to the *C. glutamicum* ATCC 13032 genome (RefSeq NC_006958.1) with default settings for paired-end read mapping. The mapped sequence data was converted from SAM to BAM format via SAMtools v1.3 [71] and imported to ReadXplorer v.2.2 [72] for data visualization and transcripts per million (TPM) [73] calculation for each coding sequence (CDS). Differential gene expression analysis was performed based on these TPM values calculating the signal intensity value (*a*-value) as mean of TPM and the signal intensity ratio (*m*-value) by log_2_-fold change for each CDS and every comparison.

#### 2.4.4. Differential Gene Expression Cut-Off Definition

In differential gene expression analysis of reduced data sets like pooled samples, the null hypothesis, and the assumption that the majority of genes are not differentially transcribed is often applied. In this study, however, the dramatic adaptations within the triple-phase process, ranging from exponential growth under aerobic conditions to non-growth anaerobic conditions, were expected to dramatically modulate the overall gene transcription [74]. Therefore, an alternative significance level was defined based on an empirical log_2_-fold change of >1.50 and <−1.50 (fold-change of 2.80 and 0.40, respectively) and *a*-value > 1.00 to exclude results derived from very few reads.

### 2.5. Calculations

*Growth rates*. Unless stated differently growth rates (µ) in h^−1^ were calculated via linear regression in semi-logarithmic plots of the biomass concentration (OD_600_ or g CDW L^−1^) over the process time. The exponential growth phase was determined by following a coefficient of determination value (R-squared) maximization strategy.

*Yields.* Calculation of yields was achieved in general through linear regression plotting biomass or product versus substrate concentration giving the biomass/substrate yield (Y_X/S_) in g CDW per g substrate or the product/substrate yield (Y_P/S_) in mol product per mol substrate, respectively.

*Substrate consumption rates.* The biomass specific substrate consumption (q_S_) was calculated within the exponential growth phase by division of the growth rate (µ) by the biomass yield (Y_X/S_). Errors were calculated applying Gaussian error propagation.

*Carbon balance*. Calculations were performed according to Buchholz et al. [75]. For the triple-phase process, the carbon was balanced using analyses of substrate (glucose) and products (biomass, lactate, succinate, acetate, CO_2_) considering the fermentation liquid volume (10 L). The net produced biomass concentrations were determined by the correlation factor (α) mentioned above and recalculated with the published carbon content of *C. glutamicum* dry biomass of 51.4% [75]. The net produced CO_2_ was determined by the exhaust gas analysis and the volumetric CO_2_ evolution rate (Q_CO2_, C-mol L^−1^ h^−1^). The Q_CO2_ was averaged between two sequential data points and summed over the entire cultivation period. The initial supply of glucose as sole carbon source represents 100% of the overall carbon. Based on this, molar carbon fractions of the single products were calculated and are visualized in Figure A2.

*Venn diagram.* RNA-sequencing data were visualized as a three-circle-overlap in the Venn diagram format [76]. For this purpose, the software *Venn Diagram Plotter* V2.0 written by Littlefield and Monroe for the Department of Energy (PNNL, Richland, WA, USA) was used (online available at https://omics.pnl.gov/software/venn-diagram-plotter). With respect to the software, we appreciate the original funding by the W.R. Wiley Environmental Molecular Science Laboratory, a national scientific user facility sponsored by the U.S. Department of Energy’s Office of Biological and Environmental Research and located at PNNL. PNNL is operated by Battelle Memorial Institute for the U.S. Department of Energy under contract DE-AC05-76RL0 1830.

*Intracellular total RNA content*. For the samples ①–⑥ within the triple-phase process, the intracellular total RNA concentration was analyzed as part of the RNA sequencing library preparation. The totally isolated RNA was quantified after purification (*RNeasy Mini Kit*, Qiagen) using a DropSense16 Micro-Volume Spectrophotometer (Xpose dscvry) (Unchained Labs, Trinean, Pleasanton, CA, USA). Considering dilution, biomass concentrations used for lysis, CDW/OD_600_ correlation and the assumption that 1 OD_600_ corresponds to approximately 10^8^ cells, we could resolve the cellular total RNA content in fg per cell.

## 3. Results

### 3.1. The Triple-Phase Batch Fermentation

**Figure 1:**
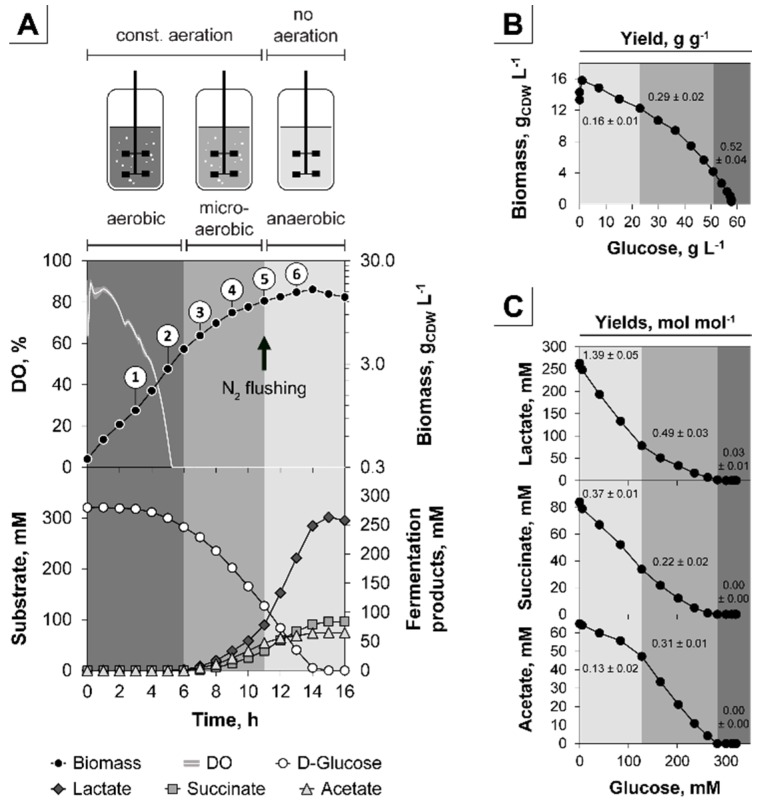
The triple-phase process with *Corynebacterium glutamicum* cultivated in CGXII + 60 g glucose L^−1^. (**A**) The 30 L bioreactor cultivation in 10 L minimal medium was realized with constant agitation (445 rpm) throughout the entire process and a gassing of 0.1 vvm within the aerobic (dark grey) and microaerobic (grey) phase. The anaerobic (light grey) phase was initiated by a stop of aeration and temporary flushing of the headspace with N_2_. Sampling for e.g., RNA-sequencing analysis is indicated with circled numbers (①, ②, ③, ④, ⑤, ⑥); (**B**) Biomass/substrate yield (Y_X/S_); (**C**) Product/substrate yields (Y_P/S_). Error bars and shaded area of the dissolved oxygen (DO) represent the standard deviation (SD) of four independent experiments.

To investigate the adaptation of *C. glutamicum* to a gradual shift from aerobiosis to anaerobiosis, we established a triple-phase batch fermentation enabling a successive transition from aerobic to anaerobic conditions via a microaerobic interface (Figure 1). Since we applied constant agitation and aeration in the first two phases of the process, the DO dropped with increasing biomass to 0% of saturation after 5 h. However, the cells continued to grow and started parallel secretion of acetate, succinate, and lactate after 6 h of cultivation. This onset of organic acid production indicated the beginning of oxygen limitation and was used to define the initiation of microaerobiosis. To establish strict anaerobic conditions, we stopped aeration and flushed the headspace of the bioreactor with N_2_ after 11 h of fermentation (Figure 1A). During the aerobic, microaerobic, and anaerobic phase this approach caused a stepwise reduction of the growth rate (µ) from 0.40 ± 0.01, 0.21 ± 0.00 to 0.09 ± 0.01 h^−1^ and of the biomass/substrate yield (Y_X/S_) from 0.52 ± 0.04, 0.29 ± 0.02 and 0.16 ± 0.01 g CDW per g glucose, respectively (Figure 1B). The biomass specific glucose consumption rate (q_S_) remained rather constant during the first two phases at 0.77 ± 0.06 and 0.72 ± 0.05 g g^−1^ h^−1^ and dropped under anaerobic conditions to 0.56 ± 0.07 g g^−1^ h^−1^ (Table 1). Although oxygen deprivation progressively increased within the microaerobic phase, a constant product/substrate yield (Y_P/S_) of 0.49 ± 0.03, 0.22 ± 0.02 or 0.31 ± 0.01 mol lactate, succinate, or acetate per mol glucose was found, respectively (Figure 1C, Table 1). The successive anaerobic phase was clearly distinguishable from microaerobiosis by a change of the related Y_P/S_ to 1.39 ± 0.05, 0.37 ± 0.01 or 0.13 ± 0.02 mol lactate, succinate, or acetate per mol glucose (Figure 1C, Table 1). For the microaerobic and anaerobic phase the overall Y_P/S_ added up to 1.02 ± 0.07 and 1.90 ± 0.08 mol fermentation products (lactate, succinate, and acetate) per mol of glucose, respectively. The carbon balance including the analyzed products (biomass, lactate, succinate, acetate, and CO_2_) with respect to the supplied carbon source glucose fully closed over the entire cultivation period to 0.99 ± 0.03 C-mol per C-mol glucose (Figure A2).

In summary, the triple-phase process shows distinct physiological characteristics in the aerobic, microaerobic, and anaerobic phase and thus represents a useful platform to analyze the global transcriptional adaptation during the installed oxygen-dependent transitions.

### 3.2. Analysis of the Transient Transcriptional Adaptation in Response to Decreasing Oxygen Availability

**Figure 2:**
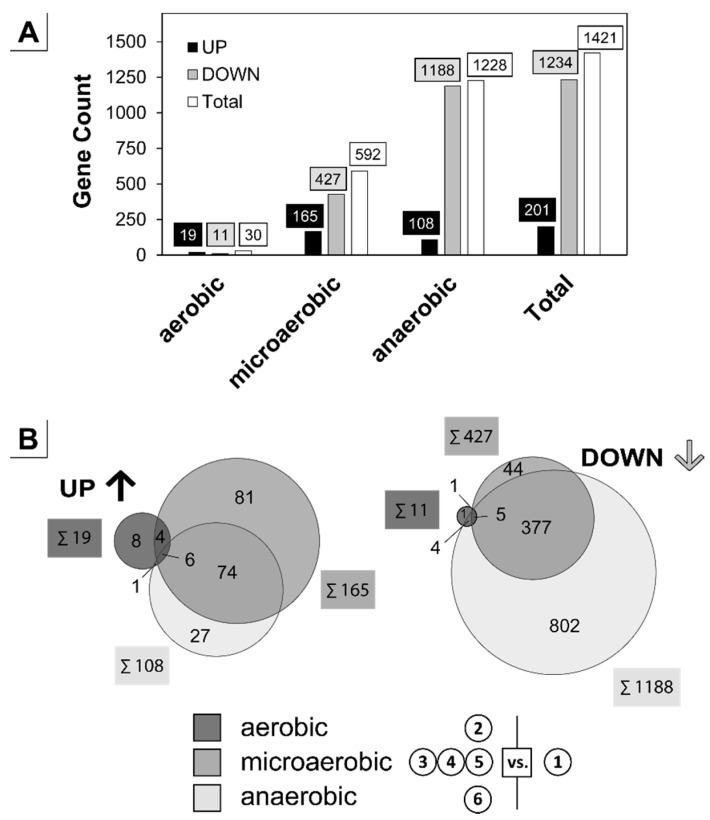
Overall transcriptional changes during the triple-phase process. RNA-sequencing analysis was conducted with a log-fold change (*m*-value, >1.50, <–1.50) and an average differential expression value (*a*-value, >1.00) cutoff with the aerobic state ① serving as reference. (**A**) Differentially expressed genes were counted within the aerobic (②), microaerobic (③, ④, ⑤) and anaerobic (⑥) phase and summed over the total process timeframe (Figure 1A). For the microaerobic phase (samples ③, ④, ⑤) an average value was calculated and allocated to up- or downregulation. (**B**) Venn diagram separated into up- and downregulated genes within the three major process phases. The sum of totally altered genes is given in boxes beside the circle of the respective phase.

To analyze the transcriptional response during the installed transitions, samples for RNA-sequencing analysis were taken under aerobic (①, ②), microaerobic (③, ④, ⑤) and anaerobic conditions (⑥) (Figure 1A). After RNA isolation, the integrity was analyzed and showed 16S/23S RNA ratios from 1.5 to 1.7 and an RNA integrity number (RIN) >8. Both indicators confirm a highly suitable RNA quality with low degradation during sampling and processing. Overall, the RNA-sequencing yielded 28,720,937 reads as assignable and unique mapping events, which were subsequently applied for calculation of log_2_ transcripts per million (log_2_TPM). Raw log_2_TPM values were analyzed by Pearson correlation and indicated a logical progression of the transcriptomic response to the increasing oxygen limitation during the fermentation process (Figure A3). The strict aerobic state of sample ① served as reference for all following analysis. Figure 2 gives an overview of all differentially transcribed genes within the respective phase. Over the entire cultivation time 1421 genes were differentially expressed compared to the aerobic reference state, representing a dramatic change of around 50% of the 3002 known protein coding genes [77]. Of this share, only 201 genes were enhanced in expression, whereas 1234 were downregulated. A clustering visualized in the Venn diagram (Figure 2B) indicates that each phase has, besides overlapping features, also unique transcriptional responses, which is in accordance with the observed phenotypic distinction in each phase.

#### 3.2.1. Central Metabolism and Amino Acid Biosynthesis

**Figure 3:**
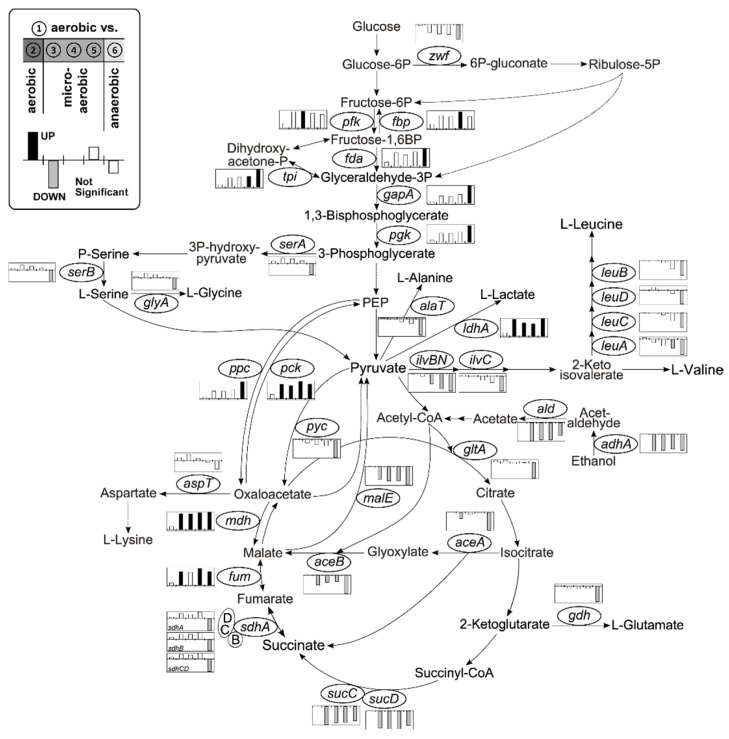
Transcriptional response to a shift from aerobiosis via microaerobiosis to anaerobiosis including genes of glycolysis, tricarboxylic acid cycle (TCA), glyoxylate shunt, oxidative pentose phosphate pathway and selected amino acid biosynthesis pathways. Column graphs represent log_2_-fold changes of enhanced (black) and reduced (grey) expression. Values outside the significance constraints (*m*-value > 1.50, < −1.50 and *a*-value > 1.00) are also shown (white). From left to right aerobiosis (②), microaerobiosis (③, ④, ⑤) and anaerobiosis (⑥) versus the aerobic reference (①; Figure 1A). Abbreviations of the given genes: *aceA* (isocitrate lyase), *aceB* (malate synthase), *adhA* (alcohol dehydrogenase), *aspT* (aspartate aminotransferase), *alaT* (alanine aminotransferase), *ald* (acetaldehyde dehydrogenase), *fbp* (fructose-1,6-bisphosphatase), *fda* (fructose-bisphosphate aldolase), *fum* (fumarate hydratase), *gapA* (glyceraldehyde-3-phosphate dehydrogenase), *gdh* (glutamate dehydrogenase), *gltA* (citrate synthase), *glyA* (serine hydroxymethyltransferase), *ilvBN* (acetohydroxyacid synthase), *ilvC* (acetohydroxyacid isomeroreductase), *ldhA* (l-lactate dehydrogenase), *leuA* (2-isopropylmalate synthase), *leuB* (3-isopropylmalate dehydrogenase), *leuCD* (3-isopropylmalate dehydratase), *malE* (malic enzyme), *mdh* (malate dehydrogenase), *pck* (phosphoenolpyruvate carboxykinase), *pfk* (6-phosphofructokinase), *pgk* (3-phosphoglycerate kinase), *ppc* (phosphoenolpyruvate carboxylase), *pyc* (pyruvate carboxylase), *sdhABCD* (succinate dehydrogenase), *serA* (phosphoglycerate dehydrogenase), *serB* (phosphoserine phosphatase), *sucCD* (succinyl-CoA synthetase), *tpi* (triosephosphate isomerase), *zwf* (subunit of the glucose-6P dehydrogenase). Graphic represents extended version to literature [81].

During the transition from aerobic to anaerobic conditions, we found a significant transcriptional activation of genes encoding glycolytic and fermentative enzymes and of the reductive branch of the tricarboxylic acid cycle (TCA) as well as a reduction of the oxidative branch of the TCA (Figure 3). Moreover, the applied experimental setup allowed to distinguish between an early transcriptional response with the onset of microaerobiosis (upregulated e.g., *ldhA*, *mdh*, *pck*; downregulated e.g., *adhA*, *ald*, *sucCD*, *malE*) and a late response upon strict anaerobiosis (e.g., *sdhABCD*, *gdh*, *gltA*). Already with the initiation of microaerobiosis, the *adhA* and *ald* gene of ethanol catabolism as well as the *sucCD* genes encoding succinyl-CoA synthetase of the oxidative branch of the TCA genes were strongly repressed with log_2_-fold changes of −9.13 (~562-fold), −5.97 (~62-fold) and −5.32 (~40-fold), respectively. Furthermore, expression of the *ldhA* (4.34; ~20-fold) gene and the *mdh* (3.20; ~9-fold) and *fum* (1.56; ~3-fold) genes responded immediately to oxygen-limitation with increased expression coinciding with the start of organic acid secretion (Figure 1 and Figure 3). It is surprising that the cluster *sdhABCD* encoding succinate dehydrogenase is reduced in transcription upon anaerobiosis by up to 5-fold, even though succinate is produced during fermentation.

**Figure 4:**
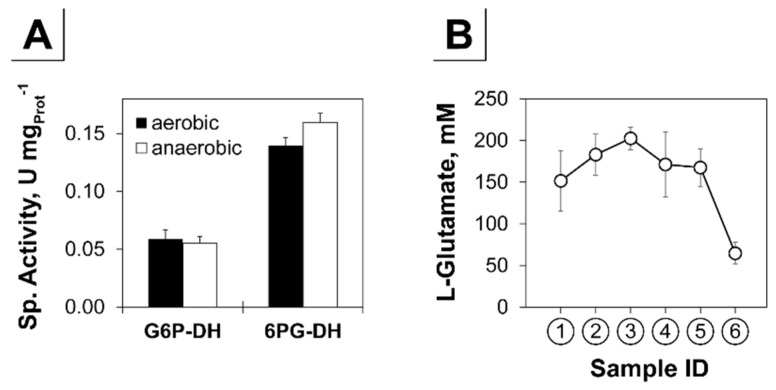
(**A**) Specific activities of the glucose-6P dehydrogenase (G6P-DH) and 6P-gluconate dehydrogenase (6PG-DH) in U per mg total protein. (**B**) Intracellular l-glutamate analysis in samples taken during the triple-phase process [aerobic (①, ②), microaerobic (③, ④, ⑤), anaerobic conditions (⑥); Figure 1A]. Error bars represent SD of three (**A**) or four (**B**) independent experiments.

While growing aerobically on glucose about 69% of the carbon flux is directed to the pentose phosphate pathway (PPP) and only 5% enter the PPP under anaerobic conditions in *C. glutamicum* [78,79]. The key enzymes of the oxidative branch of the PPP are glucose-6P dehydrogenase (G6P-DH), encoded by the genes *zwf* and *opcA*, and the 6P-gluconate dehydrogenase (6PG-DH), encoded by *gnd* [64]. Expression of the *opcA* and *gnd* genes was not changed significantly throughout the cultivation (not shown) and the *zwf* gene showed a 3-fold reduced transcription towards anaerobiosis (Figure 3). However, specific activities of the G6P-DH and 6PG-DH under aerobic exponential growth or non-growing anaerobic conditions were not substantially changed (Figure 4A) indicating a prevalence of metabolic control of the flux into the PPP (e.g., by NADPH) as proposed by Radoš et al. [79].

Expression of genes encoding amino acid biosynthetic enzymes (for example l-glutamate, l-serine, l-glycine, l-alanine, and branched chain amino acids) was rather unaffected during microaerobiosis but decreased under anaerobic conditions (Figure 3). In contrast, transcription of *aspT* encoding aspartate amino transferase connecting TCA and synthesis of l-aspartate and its derived amino acids was not affected significantly on transcriptional level in response to oxygen limitation. Since l-glutamate is the major amino donor for amino transferase reactions [80], we analyzed the intracellular l-glutamate concentrations (Figure 4B). We found a rather constant level of 167 ± 30 mM under aerobic (average of ①, ②) and 180 ± 22 mM under microaerobic (average of ③, ④, ⑤) conditions. Under subsequent anaerobic conditions (⑥), the concentration dropped by about 70% to 65 ± 13 mM compared to aerobiosis. Accordingly, with the onset of anaerobiosis, we observed 4-fold reduced transcription of the *gdh* gene encoding l-glutamate dehydrogenase (Figure 3) which, together with the strongly decreased intracellular l-glutamate concentrations, indicates a probable shortage of this amino group donor (additionally) mitigating overall amino acid synthesis.

#### 3.2.2. Respiratory Chain and Energy Metabolism

**Figure 5:**
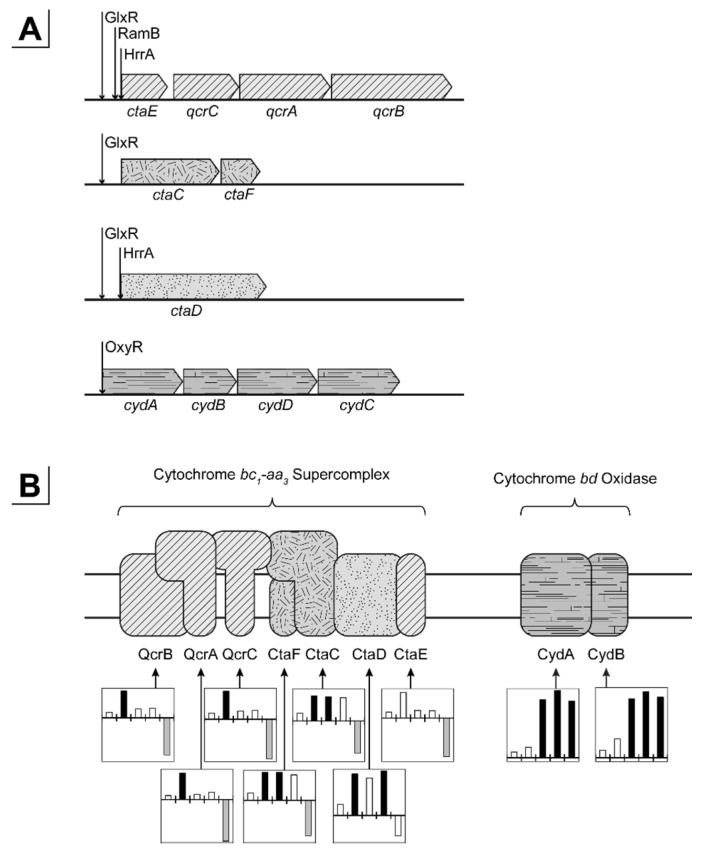
Transcriptional response of the cytochrome *bc_1_*-*aa_3_* and cytochrome *bd* oxidase to altering oxygen availabilities. (**A**) Genetic organization and operon structures. Binding of the transcriptional regulators GlxR, RamB, HcrA and OxyR is indicated. (**B**) Schematic organization of the cytochrome oxidases in the cytoplasmic membrane. Column graphs represent log_2_-fold changes of enhanced (black) and reduced (grey) expression. Open columns are values outside the significance constraints (*m*-value > 1.50, < −1.50 and *a*-value > 1.00). From left to right aerobiosis (②), microaerobiosis (③, ④, ⑤), and anaerobiosis (⑥) versus the aerobic reference (①; Figure 1A). Scaling of the graphs is variable. Shading links genes to proteins. Graphic A and B based on the online tool *CoryneRegNet* and Bott and Niebisch, respectively [86,87].

*Corynebacterium glutamicum* possesses the two oxygen dependent terminal oxidases cytochrome *bc*_1_-*aa*_3_ supercomplex and cytochrome *bd* oxidase with low and high oxygen affinity, respectively [82,83,84]. Transcription of cytochrome *bd* oxidase was induced (~14-fold) with the transition to microaerobiosis and remained high during complete anaerobic conditions, whereas the transcripts of cytochrome *bc*_1_-*aa*_3_ supercomplex decreased significantly (~4-fold) with the onset of complete anaerobic conditions (Figure 5). We found a continuously reduced expression of the type II NADH dehydrogenase encoded by *ndh* reaching a maximal and significant log_2_-fold change of −2.83 (~7.1-fold down) under anaerobic conditions. Physiologically, this membrane-bound enzyme transfers electrons from NADH to the menaquinone pool without pumping protons [85]. Since menaquinol cannot be reoxidized under oxygen limiting conditions, NAD^+^ is regenerated by the formation of reduced organic acids (i.e., lactate and succinate) and energy conservation in the fermentative metabolism is achieved by substrate level phosphorylation. Accordingly, the enhancement of oxygen deprivation led to reduced transcription of the entire *atpBEFHAGDC* operon encoding H^+^-ATPase with an average log_2_-fold change of −2.99 ± 0.44 (~7.9-fold down) under strict anaerobic conditions.

#### 3.2.3. Translation, Transcription and Replication

Under anaerobic conditions about 41% of the entire protein coding genes showed reduced transcription by a globally averaged log_2_-fold change of −2.34 ± 1.46 (~5.1-fold down). This general trend follows the reduction of the growth rate in the course of the fermentation and is accompanied by reduced transcription of genes related to basic cellular functions such as translation, transcription and replication, which were analyzed here according to clusters of orthologous genes (COG) [88,89]. Indeed, about 80% (66 of 81) of all structural ribosomal genes (selected based on Martín et al. [90] and extended with CoryneRegNet [87,90]) were reduced in expression upon anaerobiosis. This also affects the majority of the translation COG J class, where 90 of 133 genes are on average less transcribed with a log_2_-fold change of −2.99 ± 1.00 (~7.9-fold down, not shown) under oxygen deprivation. For the transcription COG K class 30% of the genes (54 of 161) were analogously altered in transcription (log_2_-fold change −2.07 ± 0.78; ~4.2-fold down; not shown). Also, in the COG L class of replication associated genes, around 40% (54 of 135) were downregulated by −1.99 ± 0.96 (~4.0-fold down; not shown). Among those are also structural genes of the DNA polymerase III (such as *dnaE1*, *dnaE2*, cg2576, and others).

**Figure 6:**
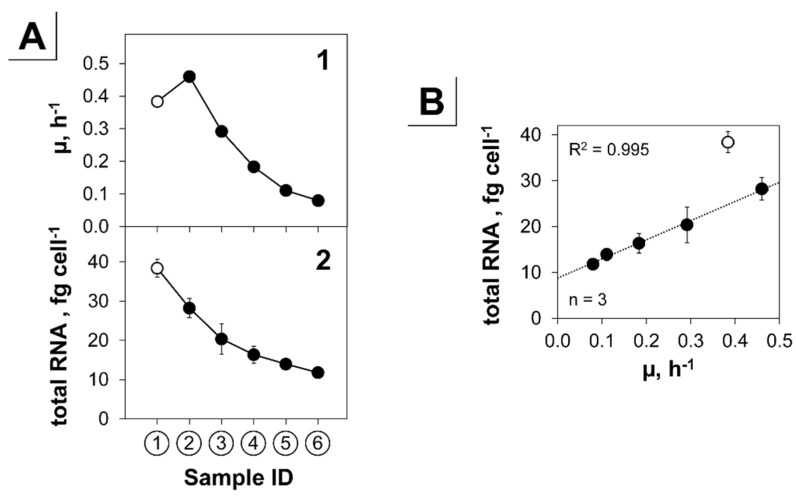
Correlation of total RNA content and growth rate within the triple-phase process. (**A**) The growth rate (µ, **1**) and the total RNA per cell (**2**) is depicted from left to right for the process phases: aerobiosis (①, ②), microaerobiosis (③, ④, ⑤), and anaerobiosis (⑥; Figure 1A). (**B**) Direct correlation of the total RNA content and the growth rate. Linear regression was calculated neglecting the first sampling point (①, open circle). Error bars represent SD of a triplicate experiment.

It is known that the growth rate influences the global expression profile [74] and concentrations of cellular components such as DNA, total RNA or cell size [91]. For each sampling point (①–⑥) in the triple-phase process, we determined the total RNA concentration per cell (Figure 6). Up to 99% of total RNA in *C. glutamicum* is rRNA [92]. Therefore, the following analysis primarily mirrors the rRNA content. We found a clear linear correlation of the RNA content and the growth rate (Figure 6B). Extrapolation of this correlation curve to zero growth (µ = 0 h^−1^) revealed a minimal total cellular RNA concentration of 8.8 ± 0.7 fg per cell, which is close to the minimal total RNA concentration of 10.8 fg per cell in *E. coli* (own extrapolation from data of Bremer et al. [91]).

#### 3.2.4. Sigma Factors and Transcriptional Regulators

*C. glutamicum* possesses seven σ-factors, which play a key role in responding to global changes such as environmental stresses or growth arrest [93,94]. Two σ-factors namely, σ^B^ and σ^D^, were previously connected to the response of *C. glutamicum* to oxygen deprivation [95,96]. In a Δ*sigB* mutant, Ehira et al. [95] found a reduced expression of genes involved in glucose metabolism. Accordingly, we analyzed a gradual increase of *sigB* transcription (and also of glycolytic genes; Figure 3) until significant levels were reached under anaerobiosis with a log_2_-fold change of 2.10 (~4.3-fold up; Table A2). In the triple-phase process the gene *sigD* was reduced in transcription with a maximal log_2_-fold change of −2.55 (~5.9-fold down). Both transcriptional alterations of the factors σ^B^ and σ^D^ might support the bacterium’s adaptation towards oxygen limitation but also to the stationary phase. The other σ-factors did not show significant or interpretable changes in transcription (Table A2).

Numerous transcriptional profiles shown in this study represent a coordinated response towards the progression of oxygen scarcity that might, besides global changes, rely on specific regulation on the transcriptional level. Furthermore, expression of transcriptional regulators themselves can change to manifest their regulatory purpose. In *E. coli* it is known that key regulators FNR and ArcBA involved in the adaptation to altering oxygen availabilities indeed show a significantly changed transcriptional level at variation of the oxygen supply [48,51]. For example, *arcA* expression was demonstrated to increase about 4-fold towards anaerobiosis [97]. Assuming that in *C. glutamicum* a similar behavior is prevalent, we screened the known 159 genes encoding DNA-binding transcription regulators, response regulators of two-component systems, and sigma factor subunits of RNA polymerase [98] for differential expression during the triple-phase process. 34 (putative) regulators were found with reduced or enhanced transcription. From these, 18 candidates were selected with a significant response in more than one condition of aerobiosis, microaerobiosis or anaerobiosis (Table A3). To analyze a possible involvement of the selected regulators in the oxygen-dependent adaptation, we deleted the entire open reading frame (ORF) of each respective gene (for more information see appendix). Although the transcriptional redox-sensor OxyR was not differentially expressed in a significant manner throughout the triple-phase process, we also deleted the coding region of this candidate. OxyR is known to sense the intracellular redox potential, however, only towards oxidative stress such as H_2_O_2_ exposure [99,100] but is also involved in the regulation of the cytochrome *bd* oxidase expression (Figure 5A) [100].

Each single mutant was cultivated in shaking flasks without baffles to ensure limiting oxygen availability (for further information see appendix). Upon deletion of mandatory regulators that coordinate the transcriptional response to oxygen scarcity, we expected to observe a specifically hampered growth phenotype. Similar findings were published by Kabus et al. [101] for *C. glutamicum* upon deletion of the cytochrome *bd* oxidase in shaking flask experiments. As a reference, we cultivated *C. glutamicum* WT in shaking flasks with and without baffles (Figure A4A). Within the first 6 h of cultivation no remarkable difference in proliferation was found with growth rates of 0.41 ± 0.01 h^−1^ or 0.38 ± 0.01^−1^, respectively. In the ongoing experiment, however, oxygen supply became critical in the flask without baffles. Over the entire cultivation, growth rates reached 0.41 ± 0.01 h^−1^ or 0.34 ± 0.01^−1^ for the baffled and unbaffled flasks. Growth significantly ceased in flasks without baffles and resulted in an enhanced fermentative phenotype indicated by a drastically reduced pH compared to the baffled system (Figure A4A). Besides *C. glutamicum* Δ*ramB* and Δ*oxyR* no other deletion strain showed a remarkable growth phenotype (not shown). *C. glutamicum* Δ*ramB* was generally reduced in proliferation (µ = 0.29 h^−^^1^) as known from previous studies [102]. This effect, however, could not be allocated to the oxygen deprivation. *C. glutamicum* Δ*oxyR* showed only slightly reduced growth in baffled flasks compared to the WT (Figure A4B). In contrast, hampered growth was observed in flasks without baffles in comparison to the WT starting at 8 h of cultivation (Figure A4C) indicating that OxyR might be involved in the coordination of *C. glutamicum*’s adaptation towards anaerobiosis.

## 4. Discussion

**Figure A1:**
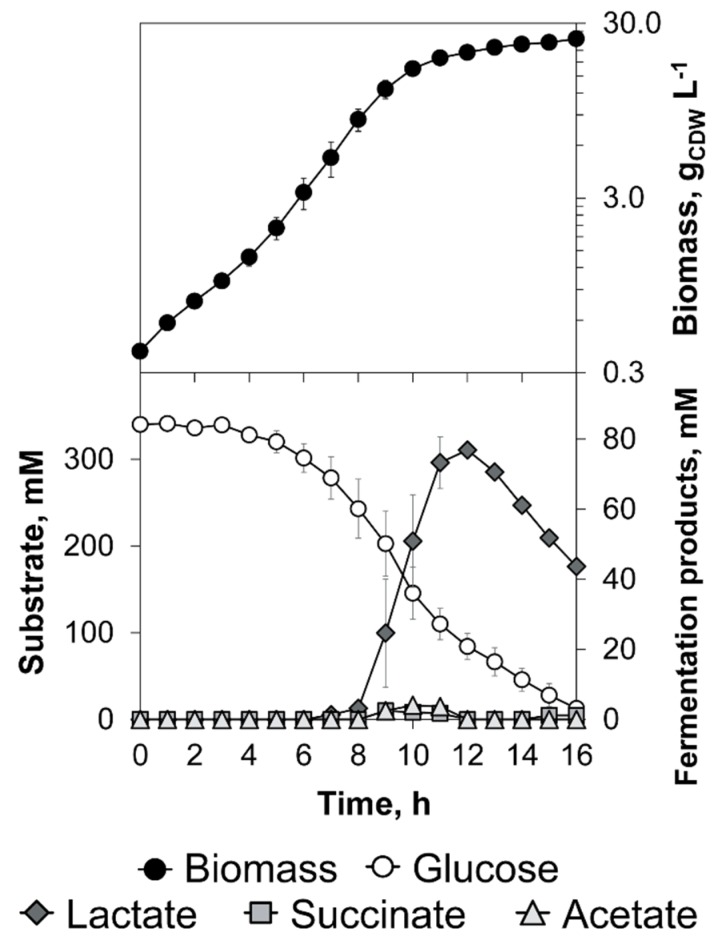
Shaking flask cultivation of *C. glutamicum* WT in CGXII + 60 g glucose L^−1^. Error bars represent the SD of five independent experiments.

**Figure A2:**
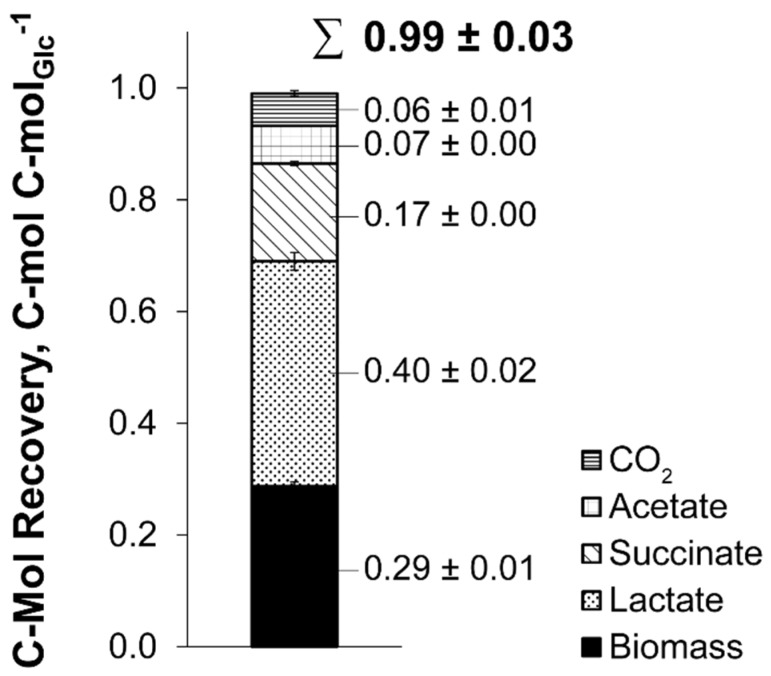
Carbon balance of the triple-phase process. Analyzed products (biomass, lactate, succinate, acetate and CO_2_) were balanced with respect to glucose as sole carbon source over the entire cultivation period of 0–16 h (Figure 1A). Error bars represent the SD of four independent experiments.

**Figure A3:**
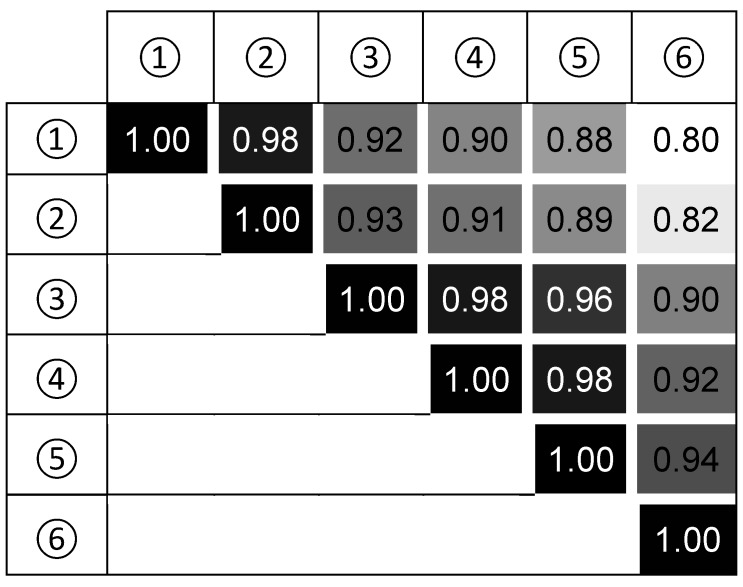
Pearson correlation of RNA-sequencing data. The log_2_TPM values within the entire raw RNA-sequencing data were correlated (no significance constraints applied). Sample IDs of the triple-phase process from left to right aerobiosis (①, ②), microaerobiosis (③, ④, ⑤) and anaerobiosis (⑥).

To analyze the transient adaptation of *C. glutamicum* to steadily decreasing oxygen-availability, we established the triple-phase process depicting transitions from aerobiosis to anaerobiosis traversing microaerobiosis. As shown, the three phases were distinguishable and clearly bordered by the respective µ, Y_X/S_ and Y_P/S_ values (Figure 1). This allowed to define the start of microaerobiosis by the changing physiological state of the cells (i.e., initiation of organic acid production as a result of oxygen-limitation) rather than by a difficult detection of close-to-zero oxygen concentrations. Interestingly, the microaerobic environment allows *C. glutamicum* to perform aerobic respiration and fermentation during growth in parallel. Such physiological conditions can typically also be found in shaking flasks with high initial sugar concentrations (Figure A1) and the observed oxygen limitation may be more pronounced in the absence of baffles or at low shaking speed additionally potentiating pH fluctuations in this system. Also, taking the observed transcriptional alterations with the onset of microaerobiosis into account (Figure 2), these results emphasize the drawback of the undefined flask systems and should be considered in designing seed trains and studies for comparative strain characterization.

**Figure 7:**
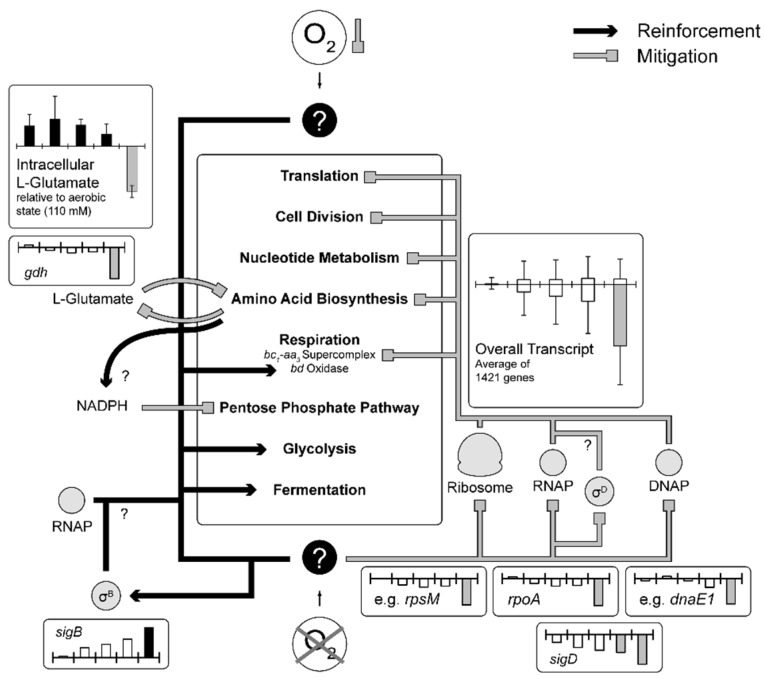
Hypothetical model of *C. glutamicum*’s response to micro- and anaerobiosis including unknown regulatory mechanisms, metabolites, or cellular signals (?). Reinforcement and mitigation is visualized by arrowheads and squares, respectively. Column graphs (with exception of intracellular l-glutamate titers) represent log_2_-fold changes of enhanced (black) and reduced (grey) expression. Open columns are values outside the significance constraints (*m*-value > 1.50, <−1.50 and *a*-value > 1.00). From left to right aerobiosis (②), microaerobiosis (③, ④, ⑤) and anaerobiosis (⑥) versus the aerobic reference (①; Figure 1A). Scaling of these graphs is variable. Intracellular l-glutamate pools are depicted relatively to aerobic intracellular titers analogously to differential expression column graphs. Error bars represent SD. Abbreviations: RNAP, RNA polymerase; DNAP, DNA polymerase.

**Figure A4:**
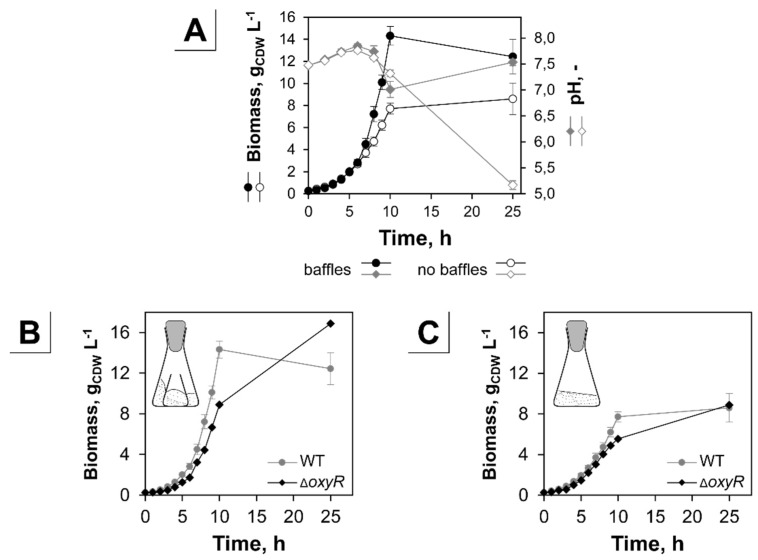
Cultivation of *C. glutamicum* WT and Δ*oxyR* under progressing oxygen limitation in shaking flasks with baffles or without baffles in CGXII + 40 g glucose L^−1^ as sole carbon source. (**A**) WT cultivation in baffled (filled symbols) and unbaffled (open symbols) shaking flasks. (**B**,**C**) Cultivation of *C. glutamicum* Δ*oxyR* in shaking flasks with baffles (**B**) and without baffles (**C**). In both graphs the WT cultivation is shown in grey (circles). Error bars show SD of four independent cultivations of the *C. glutamicum* WT strain. For *C. glutamicum* Δ*oxyR* (black, diamonds) the average of two comparable independent cultivations is depicted.

The applied experimental setup allowed to unravel an early transcriptional response with the onset of microaerobiosis and a late response as a result of complete anaerobic conditions (Figure 7). Initiation of microaerobiosis led to a transcriptional activation of genes belonging to glycolysis, the reductive branch of the TCA and fermentation in accordance to the observed formation of organic acids. Also, transcription of genes encoding high-affinity cytochrome *bd* oxidase were induced (~14-fold) in response to microaerobiosis and remained high during complete anaerobic conditions, whereas transcripts of cytochrome *bc*_1_-*aa*_3_ supercomplex decreased significantly (~4-fold) not until the onset of complete anaerobic conditions (Figure 5). The transcriptional alterations are in accordance with previous studies but were so far due to the experimental setup attributed to complete anaerobic conditions [30,95]. Interestingly, the two genes *ctaA* and *ctaB* encoding newly proposed heme *o* and *a* synthases [103], which may contribute to cytochrome oxidase assembly, show similar transcriptional patterns as the cytochrome *bd* oxidase genes (not shown). Both genes are transcribed in two separate operons [92]. Presumably, these two operons and the *cydABCD* operon are activated by similar regulatory mechanisms responding to the microaerobic environment. Toyoda and Inui [103] proposed the sigma factor σ^C^ to be involved in the described regulation since overexpression of σ^C^ enhanced expression of the *cydABCD*, *ctaA* and *ctaB* genes and also reduced transcription of *ctaE*-*qcrCAB*. However, in our experiments σ^C^ was not significantly altered in transcription over the entire cultivation time (Table A2). It should be noted that transcriptional regulation of the cytochrome *bc*_1_-*aa*_3_ supercomplex genes also relies on the master regulators GlxR and RamB and on the putative two component system HrrA [87,98]. Furthermore, expression control of the cytochrome *bd* oxidase involves the bacterial redox sensor OxyR [99,100,104]. Whereas deletion of *ramB* did not reveal an oxygen-dependent growth phenotype, deletion of *oxyR* slightly hampered growth (Figure A4) indicating an involvement of OxyR under oxygen limitation. However, the observation might be solely explained by a shortage of cytochrome *bd* oxidase as a result of the *oxyR* deletion since a cytochrome *bd* oxidase-deficient *C. glutamicum* strain shows similar growth phenotype [101]. σ^B^ and σ^D^ were previously shown to be involved in the adaptation to oxygen deprivation [95,96] and a *sigB* mutant showed reduced expression of glycolytic genes [95]. In our study *sigB* as well as genes of glycolysis (Figure 3) steadily increased until significant levels were reached under complete anaerobic conditions, whereas expression of *sigD* decreased with increasing oxygen limitation (Table A2). In contrast, a *sigD* deletion mutant was unable to grow under low oxygen concentrations [96] and, more recently, Taniguchi et al. [105] proposed a regulatory role of σ^D^ in the maintenance of the cell wall integrity. On the one hand it might be concluded that σ^B^ and σ^D^ support *C. glutamicum*’s adaptation towards oxygen limitation, on the other hand the transcriptional alterations of both sigma factors might just reflect the response to the successive growth rate reduction that comes along with increasing oxygen limitation (Figure 1A and Figure 6A). Interestingly at the initiation of microaerobiosis, we also observed a remarkable reduction (~20-fold) of the *sucC* and *sucD* transcripts, whereas the other transcripts of the oxidative branch remained rather constant (Figure 3). This indicates an oxygen-related transcriptional control of the oxidative TCA on the level of succinyl-CoA synthase activity.

With the onset of strict anaerobiosis, 41% of the entire protein coding sequences of *C. glutamicum* were significantly reduced in expression compared to the aerobic reference state. Besides the already under microaerobic conditions observed in the transcriptional activation of genes coding for enzymes of glycolysis, fermentation, cytochrome *bd* oxidase and the reductive branch of the TCA (Figure 3), we found significantly reduced transcript levels of genes of amino acid biosynthesis, translation, transcription, replication, cell division and nucleotide metabolism (Figure 7). However, a significant transcriptional alteration of genes in the PPP was not observed and also specific activities of the G6P-DH and 6PG-DH under aerobic exponential growth and non-growing anaerobic conditions were identical (Figure 4A). This hints to metabolically controlled influx of PPP leading to the observed strong reduced flux into the PPP under complete anaerobic conditions [79]. We found that intracellular l-glutamate concentrations and transcript levels of *gdh* were strongly reduced towards anaerobiosis (Figure 4B), which might result in shortage of the major amino group donor for final aminotransferase reactions. This strategy would ensure an adjustment of the overall amino acid availability to coordinate the growth cessation as a result of the anaerobic environment and moreover may result in an increased intracellular NADPH/NADP^+^ ratio. This in turn represents a potent inhibitor of G6P-DH and 6PG-DH [64] and may corroborate the hypothesis of a demand driven control of the flux into the PPP.

As stated above, with the beginning of complete anaerobic conditions, we observed a drastic reduction of 41% of the entire protein coding sequences, which might be coordinated on the level of basic cellular functions. Accordingly, we found a significant transcriptional reduction of the COG classes transcription, translation, and replication. Key structural genes of these fundamental mechanisms were transcriptionally hampered and could thus adjust the metabolism to anaerobiosis and the impending growth arrest (Figure 7). This is reflected by changes of the total intracellular RNA content, which correlated linearly with the growth rate reduction to a hypothetical minimal level of 8.8 ± 0.7 fg per cell.

To identify key regulators involved in the adaptation to altering oxygen availability, we deleted 19 genes encoding (putative) regulators. With exception of the *C. glutamicum* Δ*oxyR* mutant (discussed above), none of the mutants showed altered growth kinetics under oxygen restricted conditions. Since homologs to known oxygen sensing and regulating (e.g., ArcA/B, FNR) proteins are not annotated in the proteome of *C. glutamicum*, the regulatory mechanisms still remain puzzling.

In summary, the presented analysis provides a deep insight into the transient transcriptional adaptation to increasing oxygen-limitation and discloses the coordination of aerobic respiration and fermentation during growth and the strong reduction of general cellular functions as a response to strict anaerobic conditions to finally prime the metabolism for non-growing conditions. The generated transcriptomic data for *C. glutamicum* WT (wild type) provide a sound basis for future research and development and is especially of interest for designing zero-growth production processes.

## Figures and Tables

**Table 1:** Overview of the growth rate (µ), the biomass/substrate yield (Y_X/S_), biomass specific glucose consumption rate (q_S_) and the product/substrate yield (Y_P/S_) for the aerobic, microaerobic, and anaerobic condition of triple-phase process (Figure 1A). Errors represent the standard deviation (SD) of four independent experiments.

Phase	µ, h^−1^	Y_X/S_, g g^−1^	q_S_, g g^−1^ h^−1^	Y_P/S_, mol mol^−1^		
				Lactate	Succinate	Acetate
aerobic	0.40 ± 0.01	0.52 ± 0.04	0.77 ± 0.06	0.03 ± 0.01	0.00 ± 0.00	0.00 ± 0.00
microaerobic	0.21 ± 0.00	0.29 ± 0.02	0.72 ± 0.05	0.49 ± 0.03	0.22 ± 0.02	0.31 ± 0.01
anaerobic	0.09 ± 0.01	0.16 ± 0.01	0.56 ± 0.07	1.39 ± 0.05	0.37 ± 0.01	0.13 ± 0.02

## Appendix A

### Deletion of Transcriptional Regulators

#### Standard Molecular Biology Methods

Isolation and purification of plasmids and PCR fragments was performed after manufacturers’ instructions of E.Z.N.A.^®^ Plasmid Mini Kit I (Omega Bio-tek, Inc., Norcross, GA, USA) and NucleoSpin^®^ Gel and PCR Clean-up (Macherey-Nagel GmbH & Co. KG, Düren, Germany), respectively. Chromosomal DNA was extracted following the supplied protocol of DNeasy Blood & Tissue Kit (Qiagen).

#### Cloning of Plasmids and Deletion of Putatively Oxygen Responsive Regulators

Selected transcriptional regulators deduced from RNA-sequencing of the triple-phase process were deleted form the chromosome of *C. glutamicum*. For markerless deletion of genes encoding selected transcriptional regulators in the chromosome of *C. glutamicum*, we used the mobilizable and integrative vector pK19*mobsacB* [106].

In brief, a plasmid was assembled (1), transferred into *E. coli* (2), verified by sequencing (3), introduced into *C. glutamicum* (4) and rare double-crossover events for gene deletion selected (5). Details are explained in the following:

1. *Plasmid assembly*. The plasmid pK19*mobsacB* was linearized by restriction with HindIII/NheI, PstI/NheI, BamHI/NheI or BamHI/EcoRI, as given in Table A1, following general protocols of Thermo Fisher Scientific Inc. (Waltham, MA, USA). Cloning was performed based on the isothermal assembly principle [107]. The experimental procedure was rooted on published recommendations [108]. DNA fragments were amplified with designed primers via polymerase chain reaction (PCR) [109,110] in a Biometra TAdvanced thermocycler (Biometra GmbH, Göttingen, Germany) and applying Phusion Hot Start II HF DNA Polymerase (Thermo Fisher Scientific Inc., Waltham, MA, USA). Oligonucleotides were manufactured by the biomers.net GmbH (Ulm, Germany). Adjacent fragments granted ≥15 bps homologous overlaps by specifically designed oligonucleotides (Table A1). Where fragment size varied dramatically, overlap extension PCR [111] was used to approximate fragment size and to lower total fragment number prior to assembly. Deletion plasmids pJULΔcg3303, pJULΔcg2320, pJULΔcg2965, pJULΔcg2746, pJULΔ*sutR*, pJULΔcg1327, pJULΔ*znr*, pJULΔ*zur*, pJULΔ*farR*, pJULΔ*ripA*, pJULΔcg2648, pJULΔ*iclR*, pJULΔ*cspA*, pJULΔ*rbsR*, pJULΔ*genR*, pJULΔcg0150 and pJULΔ*mmpLR* harbor a Flank1 and a Flank2 of >500 bp homology to up- or downstream regions of the targeted regulator. Flank1 and Flank2 were amplified from the *C. glutamicum* chromosome with respective primer pairs Δ*-1/Δ*-2 and Δ*-3/Δ*-4 (asterisk stands for targeted gene; Table A1) and assembled with linearized pK19*mobsacB*.
2. *Transformation of E. coli*. Electrocompetent *E. coli* DH5α were manufactured and transformation with the above described isothermal assembly batches or pK19Δ*ramB* was achieved according to literature [112].
3. *Sequencing*. Sequencing of plasmids with the primers pK19seqfw and pK19seqrv was conducted by the GATC Biotech AG (Konstanz, Germany).
4. *Transformation of C. glutamicum*. Electrocompetent *C. glutamicum* were produced as described in literature [113]. For transformation, the plasmids were isolated from *E. coli* and transferred to *C. glutamicum* pursuing the protocols available in literature [112,114]. An additional heat shock was implemented after electroporation as recommended previously [115].
5. *Deletion selection and verification*. Selection of rare double-crossover events for gene deletion was conducted as described elsewhere [116]. Markerless deletion of the regulators via pJULΔ* and pK19Δ*ramB* were verified through colony PCR (Taq DNA Polymerase S, Genaxxon BioScience GmbH, Ulm, Germany) using the outside primer pair Δ*-1/Δ*-4 or ΔramB1/ΔramB2. Bacterial strains, cloned plasmids and applied oligonucleotides are given in Table A1.

**Table A1:** Bacterial Strains, plasmids, and oligonucleotides.

Strain, Plasmid, or Oligo-Nucleotide	Relevant Characteristics or Sequence	Source, Reference or Purpose
**Strains**		
*Escherichia coli* DH5α	F^-^ Φ80*lacZ*ΔM15 Δ(*lacZYA-argF*) U169 *endA1 recA1 hsdR17* (rk^−^, mk^+^) *supE44 thi-1 gyrA96 relA1 phoA*	[117]
*C. glutamicum* Δ*oxyR*	Markerless deletion of OxyR (cg2109)	[100]
*C. glutamicum* Δcg3303	Markerless deletion of cg3303 by homologous recombination with pJULΔcg3303	This study
*C. glutamicum* Δcg2320	Markerless deletion of cg2320 by homologous recombination with pJULΔcg2320	This study
*C. glutamicum* Δcg2965	Markerless deletion of g2965 by homologous recombination with pJULΔcg2965	This study
*C. glutamicum* Δcg2746	Markerless deletion of cg2746 by homologous recombination with pJULΔcg2746	This study
*C. glutamicum* Δ*sutR*	Markerless deletion SutR (cg0993) by homologous recombination with pJULΔ*sutR*	This study
*C. glutamicum* Δcg1327	Markerless deletion of cg1327 by homologous recombination with pJULΔcg1327	This study
*C. glutamicum* Δ*znr*	Markerless deletion of Znr (cg2500) by homologous recombination with pJULΔ*znr*	This study
*C. glutamicum* Δ*zur*	Markerless deletion of Zur (cg2502) by homologous recombination with pJULΔ*zur*	This study
*C. glutamicum* Δ*farR*	Markerless deletion of FarR (cg3202) by homologous recombination with pJULΔ*farR*	This study
*C. glutamicum* Δ*ripA*	Markerless deletion RipA (cg1120) by homologous recombination with pJULΔ*ripA*	This study
*C. glutamicum* Δcg2648	Markerless deletion of cg2648 by homologous recombination with pJULΔcg2648	This study
*C. glutamicum* Δ*iclR*	Markerless deletion of IclR (cg3388) by homologous recombination with pJULΔ*iclR*	This study
*C. glutamicum* Δ*cspA*	Markerless deletion of CspA (cg0215) by homologous recombination with pJULΔ*cspA*	This study
*C. glutamicum* Δ*rbsR*	Markerless deletion of RbsR (cg1410) by homologous recombination with pJULΔ*rbsR*	This study
*C. glutamicum* Δ*genR*	Markerless deletion of GenR (cg3352) by homologous recombination with pJULΔ*genR*	This study
*C. glutamicum* Δcg0150	Markerless deletion of cg0150 by homologous recombination with pJULΔcg0150	This study
*C. glutamicum* Δ*mmpLR*	Markerless deletion of MmpLR (cg1053) by homologous recombination with pJULΔ*mmpLR*	This study
*C. glutamicum* Δ*ramB*	Markerless deletion of RamB (cg0444) by homologous recombination with pK19Δ*ramB*	This study
**Plasmids**		
pK19*mobsacB*	For chromosomal integration and deletion of genetic information (*lacZ*α, RP4 *mob*, *oriV_E. coli_*, *sacB_B. subtilis_*, Kan^R^)	[106]
pJULΔcg3303	For deletion of cg3303, pK19*mobsacB*::(Flank1-Flank2), Kan^R^	This study
pJULΔcg2320	For deletion of cg2320, pK19*mobsacB*::(Flank1-Flank2), Kan^R^	This study
pJULΔcg2965	For deletion of cg2965, pK19*mobsacB*::(Flank1-Flank2), Kan^R^	This study
pJULΔcg2746	For deletion of cg2746, pK19*mobsacB*::(Flank1-Flank2), Kan^R^	This study
pJULΔ*sutR*	For deletion of *sutR* (cg0993), pK19*mobsacB*::(Flank1-Flank2), Kan^R^	This study
pJULΔcg1327	For deletion of cg1327, pK19*mobsacB*::(Flank1-Flank2), Kan^R^	This study
pJULΔ*znr*	For deletion of *znr* (cg2500), pK19*mobsacB*::(Flank1-Flank2), Kan^R^	This study
pJULΔ*zur*	For deletion of *zur* (cg2502), pK19*mobsacB*::(Flank1-Flank2), Kan^R^	This study
pJULΔ*farR*	For deletion of *farR* (cg3202), pK19*mobsacB*::(Flank1-Flank2), Kan^R^	This study
pJULΔ*ripA*	For deletion of *ripA* (cg1120), pK19*mobsacB*::(Flank1-Flank2), Kan^R^	This study
pJULΔcg2648	For deletion of cg2648, pK19*mobsacB*::(Flank1-Flank2), Kan^R^	This study
pJULΔ*iclR*	For deletion of *iclR* (cg3388), pK19*mobsacB*::(Flank1-Flank2), Kan^R^	This study
pJULΔ*cspA*	For deletion of *cspA* (cg0215), pK19*mobsacB*::(Flank1-Flank2), Kan^R^	This study
pJULΔ*rbsR*	For deletion of *rbsR* (cg1410), pK19*mobsacB*::(Flank1-Flank2), Kan^R^	This study
pJULΔ*genR*	For deletion of *genR* (cg3352), pK19*mobsacB*::(Flank1-Flank2), Kan^R^	This study
pJULΔcg0150	For deletion of cg0150, pK19*mobsacB*::(Flank1-Flank2), Kan^R^	This study
pJULΔ*mmpLR*	For deletion of *mmpLR* (cg1053), pK19*mobsacB*::(Flank1-Flank2), Kan^R^	This study
pK19Δ*ramB*	For deletion of *ramB* (cg0444), based on pK19*mobsacB*, Kan^R^	[102]
**Oligonucleotides**	**5′ → 3′**	
pK19seqfw	TAATGCAGCTGGCACGAC	Fw Sequencing primer pK19*mobsaB* derivatives [118]
pK19seqrv	TAATGGTAGCTGACATTCATCCG	Rv Sequencing primer pK19*mobsaB* derivatives
Δcg3303-1	GAAACAGCTATGACCATGATTACGCCAAGCTTGCCGTCGCAGCACATTGG	Fw primer Flank1 in pJULΔcg3303 (pK19*mobsacB*, HindIII)
Δcg3303-2	CCCCAGTACCATGCAGCTG	Rv primer Flank1 in pJULΔcg3303 (Flank2)
Δcg3303-3	CTTCGCGCAGCTGCATGGTACTGGGGATCATTATCTCCTGTTCTTGAACTGAAG	Fw primer Flank2 in pJULΔcg3303 (Flank1)
Δcg3303-4	GTGCTTGCGGCAGCGTGAAGCTAGCCCGAGTTCTCCCGTCAGC	Rv primer Flank2 in pJULΔcg3303 (pK19*mobsacB*, NheI)
Δcg2320-1	AACAGCTATGACCATGATTACGCCAAGCTTGCTGCGGGGATCACTAAA	Fw primer Flank1 in pJULΔcg2320 (pK19*mobsacB*, HindIII)
Δcg2320-2	AAAGAGCTTTTCAGAACACTTGGCAAACCTCAC	Rv primer Flank1 in pJULΔcg2320 (Flank2)
Δcg2320-3	TTGCCAAGTGTTCTGAAAAGCTCTTTCATCC	Fw primer Flank2 in pJULΔcg2320 (Flank1)
Δcg2320-4	CCTGAGTGCTTGCGGCAGCGTGAAGCTAGCGCAACAGTAGATGGAGCTG	Rv primer Flank2 in pJULΔcg2320 (pK19*mobsacB*, NheI)
Δcg2965-1	ATTACGCCAAGCTTGCATGCCTGCAGGGCGCACACGTATGGGCAGA	Fw primer Flank1 in pJULΔcg2965 (pK19*mobsacB*, PstI)
Δcg2965-2	GAGAACAAAAACCGGTGCGTACCACAATAGAGTCTTAG	Rv primer Flank1 in pJULΔcg2965 (Flank2)
Δcg2965-3	TGTGGTACGCACCGGTTTTTGTTCTCAGGCGGA	Fw primer Flank2 in pJULΔcg2965 (Flank1)
Δcg2965-4	GAGTGCTTGCGGCAGCGTGAAGCTAGCCCATCGGAAATTCACTGATGTGC	Rv primer Flank2 in pJULΔcg2965 (pK19*mobsacB*, NheI)
Δcg2746-1	AACAGCTATGACCATGATTACGCCAAGCTTGCGTGGATCCTGACCTGAAG	Fw primer Flank1 in pJULΔcg2746 (pK19*mobsacB*, HindIII)
Δcg2746-2	AACCTGGGATTCCAAAATTGCACCTATATATATGGTGCAAAAC	Rv primer Flank1 in pJULΔcg2746 (Flank2)
Δcg2746-3	TAGGTGCAATTTTGGAATCCCAGGTTAGCGGGG	Fw primer Flank2 in pJULΔcg2746 (Flank1)
Δcg2746-4	GTGCTTGCGGCAGCGTGAAGCTAGCCGTCGTCGTGCTGGATGC	Rv primer Flank2 in pJULΔcg2746 (pK19*mobsacB*, NheI)
ΔsutR-1	AACAGCTATGACCATGATTACGCCAAGCTTCACAATCATGATCGCAGCGG	Fw primer Flank1 in pJULΔ*sutR* (pK19*mobsacB*, HindIII)
ΔsutR-2	TCACGGAGGAATACCTTTTACCCTCTAGAGACGACTATCAG	Rv primer Flank1 in pJULΔ*sutR* (Flank2)
ΔsutR-3	AGAGGGTAAAAGGTATTCCTCCGTGACTAGGCTAGATGACGGATCC	Fw primer Flank2 in pJULΔ*sutR* (Flank1)
ΔsutR-4	CCTGAGTGCTTGCGGCAGCGTGAAGCTAGCGCATGCGGGTGTTTGCGCGG	Rv primer Flank2 in pJULΔ*sutR* (pK19*mobsacB*, NheI)
Δcg1327-1	GCATGCCTGCAGGTCGACTCTAGAGGATCCGCTCGGCAACTGAGGTGCCC	Fw primer Flank1 in pJULΔcg1327 (pK19*mobsacB*, BamHI)
Δcg1327-2	CAAGCGGAAAGTAAAGCCCATGCTACCCAGGATATTTTC	Rv primer Flank1 in pJULΔcg1327 (Flank2)
Δcg1327-3	GTAGCATGGGCTTTACTTTCCGCTTGTTTGATCTAG	Fw primer Flank2 in pJULΔcg1327 (Flank1)
Δcg1327-4	CCTGAGTGCTTGCGGCAGCGTGAAGCTAGCCAGGTCTTCCACGTTTTCATG	Rv primer Flank2 in pJULΔcg1327 (pK19*mobsacB*, NheI)
Δznr-1	AACAGCTATGACCATGATTACGCCAAGCTTGTCCGCACGGTAACGCTTGTG	Fw primer Flank1 in pJULΔ*znr* (pK19*mobsacB*, HindIII)
Δznr-2	GTACTTCGATAGTGGGGAAGTCCTTCCGTCCTTAG	Rv primer Flank1 in pJULΔ*znr* (Flank2)
Δznr-3	GAAGGACTTCCCCACTATCGAAGTACATTTTGTGTC	Fw primer Flank2 in pJULΔ*znr* (Flank1)
Δznr-4	CCTGAGTGCTTGCGGCAGCGTGAAGCTAGCCAAGGCTATTTTTCGAAATAG	Rv primer Flank2 in pJULΔ*znr* (pK19*mobsacB*, NheI)
Δzur-1	AACAGCTATGACCATGATTACGCCAAGCTTGTCATTTTGCGGTCTTCGCG	Fw primer Flank1 in pJULΔ*zur* (pK19*mobsacB*, HindIII)
Δzur-2	ATATGTCCTTGAACGTTGATCCTCCTCAATGACAC	Rv primer Flank1 in pJULΔ*zur* (Flank2)
Δzur-3	AGGAGGATCAACGTTCAAGGACATATGAAGCTGTCGAAC	Fw primer Flank2 in pJULΔ*zur* (Flank1)
Δzur-4	CCTGAGTGCTTGCGGCAGCGTGAAGCTAGCCATCGCCGGAGTCGTCATCA	Rv primer Flank2 in pJULΔ*zur* (pK19*mobsacB*, NheI)
ΔfarR-1	AACAGCTATGACCATGATTACGCCAAGCTTGATCCTTTGGCTCGAAATCAAAAG	Fw primer Flank1 in pJULΔ*farR* (pK19*mobsacB*, HindIII)
ΔfarR-2	AAATGGGTTCACGGGTGTTCATTTTAGCCGATCTG	Rv primer Flank1 in pJULΔ*farR* (Flank2)
ΔfarR-3	TAAAATGAACACCCGTGAACCCATTTTGGTGGC	Fw primer Flank2 in pJULΔ*farR* (Flank1)
ΔfarR-4	CCTGAGTGCTTGCGGCAGCGTGAAGCTAGCCTTCCGCAGGTGGCAGGATC	Rv primer Flank2 in pJULΔ*farR* (pK19*mobsacB*, NheI)
ΔripA-1	AACAGCTATGACCATGATTACGCCAAGCTTGACCCCTATTTTCCAGGGATC	Fw primer Flank1 in pJULΔ*ripR* (pK19*mobsacB*, HindIII)
ΔripA-2	ACCTTTACTACCTATCTCATCCTCACTACAAGCAAATTT	Rv primer Flank1 in pJULΔ*ripR* (Flank2)
ΔripA-3	GTGAGGATGAGATAGGTAGTAAAGGTGTGAAAATAGTTCCTCACG	Fw primer Flank2 in pJULΔ*ripR* (Flank1)
ΔripA-4	CCTGAGTGCTTGCGGCAGCGTGAAGCTAGCGTGCCAAGGACTGCCTGGCC	Rv primer Flank2 in pJULΔ*ripR* (pK19*mobsacB*, NheI)
Δcg2648-1	AACAGCTATGACCATGATTACGCCAAGCTTGTGATCTTTGAACGGGTGTC	Fw primer Flank1 in pJULΔcg2648 (pK19*mobsacB*, HindIII)
Δcg2648-2	TTCTTTAATCTCAAAATTTAAAATTCCATAAATTTAGACAATC	Rv primer Flank1 in pJULΔcg2648 (Flank2)
Δcg2648-3	GAATTTTAAATTTTGAGATTAAAGAAGCAGCTTCTTG	Fw primer Flank2 in pJULΔcg2648 (Flank1)
Δcg2648-4	CCTGAGTGCTTGCGGCAGCGTGAAGCTAGCGTAGTGAAATTCTCCGCGCG	Rv primer Flank2 in pJULΔcg2648 (pK19*mobsacB*, NheI)
ΔiclR-1	GCATGCCTGCAGGTCGACTCTAGAGGATCCGTGTCATAGCCGAAGAGAAG	Fw primer Flank1 in pJULΔ*iclR* (pK19*mobsacB*, BamHI)
ΔiclR-2	GTCAATGAATTGCATTTGATCCGTTTTTCTAAAG	Rv primer Flank1 in pJULΔ*iclR* (Flank2)
ΔiclR-3	AAACGGATCAAATGCAATTCATTGACGTACAAAGTGATG	Fw primer Flank2 in pJULΔ*iclR* (Flank1)
ΔiclR-4	CCTGAGTGCTTGCGGCAGCGTGAAGCTAGCCGATTCAGACAGGCGGACGT	Rv primer Flank2 in pJULΔ*iclR* (pK19*mobsacB*, NheI)
ΔcspA-1	GCATGCCTGCAGGTCGACTCTAGAGGATCCGGTTACTTTTTCGGGGCCTTTTG	Fw primer Flank1 in pJULΔ*cspA* (pK19*mobsacB*, BamHI)
ΔcspA-2	TAGCAGTTAGAGCATTTGTACCTTTTCCTAATCAGGTGATG	Rv primer Flank1 in pJULΔ*cspA* (Flank2)
ΔcspA-3	AAAAGGTACAAATGCTCTAACTGCTAGCTAAAAATTCCGC	Fw primer Flank2 in pJULΔ*cspA* (Flank1)
ΔcspA-4	CGACGTTGTAAAACGACGGCCAGTGAATTCGGAAGGCTTGCTCCCACTGC	Rv primer Flank2 in pJULΔ*cspA* (pK19*mobsacB*, EcoRI)
ΔrbsR-1	GCATGCCTGCAGGTCGACTCTAGAGGATCCGACCTTCACGGGAATTGGAC	Fw primer Flank1 in pJULΔ*rbsR* (pK19*mobsacB*, BamHI)
ΔrbsR-2	ATGAAGCGCTTGTCTCCTCACCAACTTTCTGGAAG	Rv primer Flank1 in pJULΔ*rbsR* (Flank2)
ΔrbsR-3	AGTTGGTGAGGAGACAAGCGCTTCATCAGCATG	Fw primer Flank2 in pJULΔ*rbsR* (Flank1)
ΔrbsR-4	CCTGAGTGCTTGCGGCAGCGTGAAGCTAGCCAATTTCACGACCAGTCAACG	Rv primer Flank2 in pJULΔ*rbsR* (pK19*mobsacB*, NheI)
ΔgenR-1	AACAGCTATGACCATGATTACGCCAAGCTTCCACAGGGTAGGGGAGATG	Fw primer Flank1 in pJULΔ*genR* (pK19*mobsacB*, HindIII)
ΔgenR-2	GGAAAGAGTGATTATGGGGGGAATTTTCAGAGC	Rv primer Flank1 in pJULΔ*genR* (Flank2)
ΔgenR-3	AAATTCCCCCCATAATCACTCTTTCCAGATAGCG	Fw primer Flank2 in pJULΔ*genR* (Flank1)
ΔgenR-4	CCTGAGTGCTTGCGGCAGCGTGAAGCTAGCGGTCTTACGTGGAACCAAATC	Rv primer Flank2 in pJULΔ*genR* (pK19*mobsacB*, NheI)
Δcg0150-1	GCATGCCTGCAGGTCGACTCTAGAGGATCCCTCGGACTGCGGGGTGTAC	Fw primer Flank1 in pJULΔcg0150 (pK19*mobsacB*, BamHI)
Δcg0150-2	CACAATCGATGAACTCCATAACGAGAACTTAATCGAGCAAC	Rv primer Flank1 in pJULΔcg0150 (Flank2)
Δcg0150-3	TCTCGTTATGGAGTTCATCGATTGTGAGTGAGCGGTAATAATG	Fw primer Flank2 in pJULΔcg0150 (Flank1)
Δcg0150-4	CCTGAGTGCTTGCGGCAGCGTGAAGCTAGCGAAATTGTGCGAGGCCCCCG	Rv primer Flank2 in pJULΔcg0150 (pK19*mobsacB*, NheI)
ΔmmpLR-1	GCATGCCTGCAGGTCGACTCTAGAGGATCCGAACAAGACAACCTCTACATCTTCG	Fw primer Flank1 in pJULΔ*mmpLR* (pK19*mobsacB*, BamHI)
ΔmmpLR-2	AAGGAAAATGTAGAAATTGTGGCGTGTGAACCTC	Rv primer Flank1 in pJULΔ*mmpLR* (Flank2)
ΔmmpLR-3	CACGCCACAATTTCTACATTTTCCTTCAGTTCCTCGGTGC	Fw primer Flank2 in pJULΔ*mmpLR* (Flank1)
ΔmmpLR-4	CCTGAGTGCTTGCGGCAGCGTGAAGCTAGCCATTGATCGCGGCTCTGGGC	Rv primer Flank2 in pJULΔ*mmpLR* (pK19*mobsacB*, NheI)
ΔramB1	CCACGCCGGGCACCTG	Fw primer Δ*ramB* verification
ΔramB2	GGCGCGATAGTGGATTCGTG	Rv primer Δ*ramB* verification

**Table A2:** Relative differential expression of sigma factors. Description based on literature [98,103]. Column graphs represent log_2_-fold changes of enhanced (black) and reduced (grey) expression. Open columns are values outside the significance constraints (*m*-value > 1.50, <−1.50 and *a*-value > 1.00). From left to right aerobiosis (②), microaerobiosis (③, ④, ⑤) and anaerobiosis (⑥) versus the aerobic reference (①; Figure 1A). Scaling of these graphs is variable.

Gene ID	Name	Description	Rel. Diff. Expression
cg2092	*sigA*	Primary (housekeeping) sigma factor	
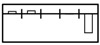
cg2102	*sigB*	Nonessential primary-like sigma factor involved in gene expression during the transition phase, under oxygen deprivation and during environmental stress responses	
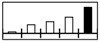
cg0309	*sigC*	Regulates expression of a branched quinol oxidation pathway	
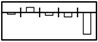
cg0696	*sigD*	ECF sigma factor probably involved in the adaptation to micro-aerobic environments	
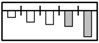
cg1271	*sigE*	ECF sigma factor involved in responses to cells surface stresses	
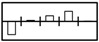
cg0876	*sigH*	ECF sigma factor controlling the heat and oxidative stress response	
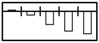
cg3420	*sigM*	ECF sigma factor controlling the expression of disulfide stress-related genes	
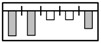

**Table A3:** Relative differential expression of putatively oxygen responsive regulators. Description based on literature [98,103]. Column graphs represent log_2_-fold changes of enhanced (black) and reduced (grey) expression. Open columns are values outside the significance constraints (*m*-value > 1.50, <−1.50 and *a*-value > 1.00). From left to right aerobiosis (②), microaerobiosis (③, ④, ⑤) and anaerobiosis (⑥) versus the aerobic reference (①; Figure 1A). Scaling of these graphs is variable.

No.	Gene ID	Name	Description	Rel. Diff. Expression
1	cg0993	*sutR*	Bacterial regulatory protein	
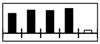
2	cg3303	*-*	Putative transcriptional regulator, PadR-family	
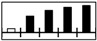
3	cg1327	*-*	Putative transcriptional regulator, Crp-family	
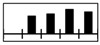
4	cg2500	*znr*	Putative transcriptional regulator, ArsR-family	
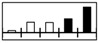
5	cg2502	*zur*	Putative transcriptional regulator, Fur-family	
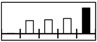
6	cg3202	*farR*	Transcriptional regulator, GntR-family	
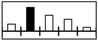
7	cg1120	*ripA*	Repressor of iron protein genes	
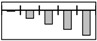
8	cg2965	*-*	Putative transcriptional regulator, AraC-family	
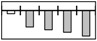
9	cg0444	*ramB*	Master regulator of carbon metabolism	
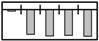
10	cg2320	*-*	Putative transcriptional regulator, ArsR-family	
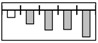
11	cg2746	*-*	Putative sugar diacid utilization regulator	
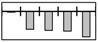
12	cg2648	*-*	Putative transcriptional regulator, ArsR-family	
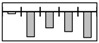
13	cg3388	*iclR*	Activator of putative hydroxyquinol pathway genes	
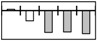
14	cg0215	*cspA*	Cold-shock protein A	
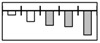
15	cg1410	*rbsR*	Repressor of ribose uptake and uridine utilization genes	
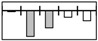
16	cg3352	*genR*	Transcriptional activator of gentisate catabolism	
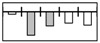
17	cg0150	*-*	Putative transcriptional regulatory protein, Fic/Doc family	
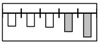
18	cg1053	*mmpLR*	Putative transcriptional regulator, TetR-family	
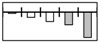
19	cg2109	*oxyR*	Hydrogen peroxide sensing regulator	
